# Porous Aromatic Framework with Tailored Binding Sites and Pore Sizes as a High‐Performance Hemoperfusion Adsorbent for Bilirubin Removal

**DOI:** 10.1002/advs.202001899

**Published:** 2020-10-25

**Authors:** Rui Zhao, Tingting Ma, Fengchao Cui, Yuyang Tian, Guangshan Zhu

**Affiliations:** ^1^ Faculty of Chemistry Northeast Normal University Changchun 130024 P. R. China

**Keywords:** adsorption, bilirubin, hemoperfusion, porous aromatic framework

## Abstract

Highly efficient removal of bilirubin from blood by hemoperfusion for liver failure therapy remains a challenge in the clinical field due to the low adsorption capacity and slow adsorption kinetics of currently used bilirubin adsorbents (e.g., activated carbon and ion‐exchange resin). Recently, porous aromatic frameworks (PAFs) with high surface areas, tunable structures, and remarkable stability provide numerous possibilities to obtain satisfying adsorbents. Here, a cationic PAF with more mesopores, named iPAF‐6, is successfully constructed via a de novo synthetic strategy for bilirubin removal. The prepared iPAF‐6 exhibits a record‐high adsorption capacity of 1249 mg g^−1^ and can adsorb bilirubin from 150 mg L^−1^ to normal concentration in just 5 min. Moreover, iPAF‐6 shows a removal efficiency of 96% toward bilirubin in the presence of 50 g L^−1^ bovine serum albumin. It is demonstrated that positively charged aromatic frameworks and large pore size make a significant contribution to its excellent adsorption ability. More notably, iPAF‐6/polyethersulfone composite fibers or beads are fabricated for practical hemoperfusion adsorption, which also show better removal performance than commercial adsorbents. This work can offer a new possibility for designing PAF‐based bilirubin adsorbents with an appealing application prospect.

## Graphical abstract

Cationic porous aromatic framework with more mesopores (iPAF‐6) is synthesized through the targeted design for highly efficient removal of bilirubin. The iPAF‐6 exhibits fast and high bilirubin uptake with a record‐high adsorption capacity of 1249 mg g^−1^. In addition, iPAF‐6/polyethersulfone composite fibers or beads show promising potential in practical hemoperfusion applications.

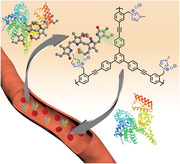

Bilirubin is the major product of hemoglobin metabolism and is transported by albumin to liver for excretion.^[^ ^1^ ^]^ The normal total bilirubin level in serum is 0.1−1.0 mg dL^−1^.^[^ ^2^ ^]^ However, people with liver failure cannot eliminate bilirubin timely, leading to the excess accumulation of bilirubin in blood, called hyperbilirubinemia.^[^ ^3^ ^]^ The extra bilirubin can cause yellow discoloration of skin and also bring about series of injuries to tissues and organs, and even result in death.^[^ ^4^ ^]^ Therefore, it has a great significance to remove extra bilirubin from blood for patients suffering from liver failure, which is also a big clinical challenge. Common extracorporeal blood purification therapies, such as hemodialysis, are unable to effectively remove bilirubin, which belongs to a protein‐bound toxin because the binding between the albumin and toxin prevents toxin from passing through the dialysis membrane.^[^ ^5^ ^]^ Currently, the most effective therapy to remove bilirubin from blood is hemoperfusion whose critical unit is the adsorbent system. Traditional bilirubin adsorbents used in clinical practice are activated carbon (AC) and exchange resin but the weak binding ability of AC and low specific surface area of exchange resin result in the unsatisfactory therapeutic efficacy. Recently, advanced porous materials, such as zirconium‐based metal‐organic frameworks (MOFs) with high specific surface area and strong binding ability, were reported for removing protein‐bound toxin from blood.^[^ ^5c^ ^]^ However, poor stability of MOFs in aqueous solutions is a major limitation to its further application, especially for blood purification. Considering the limitations and weaknesses associated with existing adsorbents,^[^ ^6^ ^]^ it is imperative to develop advanced adsorbents that features strong binding ability, high specific surface area, and excellent stability for highly efficient bilirubin removal.

Porous aromatic frameworks (PAFs) represent an important category of advanced porous materials that are constructed from various rigid aromatic building units via carbon‐carbon coupling reactions.^[^ ^7^ ^]^ The rigidness of the frameworks makes their structures porous. Owning to their large surface area, tunable porosity, ready functionality, and remarkable stability, PAFs have been widely used as adsorbents in liquid or gas, which are promising alternatives to traditional adsorbents.^[^ ^8^ ^]^ In human blood, bilirubin is binded to serum albumin with several binding sites through electrostatic interaction and/or van der Waals forces,^[^ ^9^ ^]^ which makes biological serum albumin based materials be efficient bilirubin adsorbents.^[^ ^10^ ^]^ However, the high economic cost of serum albumin limits their real usage. In view of cost‐effective aspect, targeted‐directed artificial chemical synthesis is more suitable for practical applications. Inspired by the interactions between bilirubin and albumin, we postulate that cationic PAFs are promising bilirubin adsorbents that can include the above‐mentioned interactions. The cationic frameworks can adsorb bilirubin via electrostatic interaction and the aromatic building units can capture bilirubin via van der Waals forces, leading to high binding affinity and good selectivity. Nevertheless, another issue to consider is that bilirubin has a molecular dimension of 1.94 × 0.91 × 0.67 nm (Figure S3, Supporting Information). The reported PAFs show dominating pore size distribution in micropores (<2 nm), which restricts the bilirubin diffusion during the adsorption process.^[^ ^11^ ^]^ Thus, PAFs with more mesoporous distribution are highly desired to obtain fast adsorption kinetics.^[^ ^12^ ^]^ Given that the aforementioned challenges, our endeavor in this study is to regulate the charge and pore size distribution of PAFs to construct highly efficient bilirubin adsorbents. To the best of our knowledge, the application of stable porous organic frameworks for the toxin removal from blood of patients suffering liver failure has not been reported.

In this study, two cationic PAFs (denoted as iPAF‐5 and iPAF‐6) have been synthesized using a de novo synthetic strategy involving the combination of linear cationic building unit (3‐(2,5‐dibromobenzyl)‐1‐methyl‐1H‐imidazol‐3‐ium bromide (DBMIIB)) and two different triangular linkers (1,3,5‐triethynylbenzene (TEB) and 1,3,5‐tris(4‐ethynylphenyl)benzene (TEPB)) via the Sonogashira−Hagihara cross‐coupling reaction. Then, an ion‐exchange process from Br^−^ ions to less toxic Cl^−^ ions in the imidazolium groups is carried out (**Figure** **1**). Compared with TEB, TEPB has a longer structure length, which makes iPAF‐6 have more mesoporous distributions when compared to iPAF‐5. The results demonstrate that iPAF‐6 with lager pore sizes and accessible designed adsorption sites achieves better adsorption performances toward bilirubin relative to existing adsorbents. Additionally, iPAF‐6/polymer composite beads or fibers can be obtained to add applicability for practical hemoperfusion process, which also exhibit fast and high bilirubin removal.

**Figure 1:**
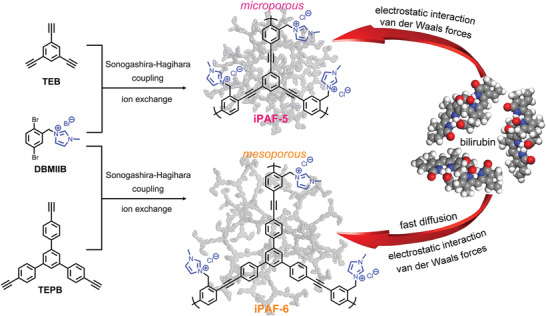
Synthetic routes of iPAF‐5 and iPAF‐6 from Sonogashira−Hagihara cross‐coupling reaction and ion‐exchange process for bilirubin adsorption.

Both iPAF‐5 and iPAF‐6 are brown powder products with microscopic spherical morphology (Figure S4, Supporting Information) and show amorphous structures (Figure S5, Supporting Information), which agrees with the previously reported porous materials from Sonogashira−Hagihara cross‐coupling reaction.^[^ ^8^, ^13^ ^]^ The Fourier transform infrared (FTIR; Figure S6, Supporting Information) spectra and solid‐state ^13^C cross‐polarization magic angle spinning (CP/MAS) nuclear magnetic resonance (NMR) (**Figure** **2**) indicate that the cross‐coupling reactions happen between DBMIIB and TEB (or TEPB), and imidazolium groups keep their integrity during the reactions. To further assess their chemical compositions, X‐ray photoelectron spectroscopy (XPS) and element analyses were also conducted. The full survey spectra and high‐resolution spectra reveal the element composition of iPAF‐5 and iPAF‐6 (Figure S7, Supporting Information). The high‐resolution N 1s spectra of both samples can be split into two peaks assigning to cationic N atoms (402.0 and 401.8 eV) and nonionic N atoms (400.6 and 400.5 eV) in the imidazolium rings, respectively (Figure S7b, Supporting Information).^[^ ^14^ ^]^ The high‐resolution C 1s spectra can be also deconvoluted into two peaks assigning to C–C (286.1 and 286.8 eV) and C–N (284.6 and 284.5 eV) carbon species (Figure S8, Supporting Information).^[^ ^15^ ^]^ Moreover, no obvious signals for Br 2p can be observed in the high‐resolution spectra which is consistent with energy‐dispersive X‐ray spectroscopy (EDS) elemental mapping data (Figure S9, Supporting Information), implying the replacement of Br^–^ with Cl^–^. Elemental analyses results show that the experimental values for C and N are lower than the theoretical values (Table S1, Supporting Information) because of the adsorbed water molecules in the frameworks. According to elemental analyses results, the contents of cationic imidazolium groups are 3.02 and 2.13 mmol g^−1^, respectively. In addition, the existence of adsorbed water can be also confirmed by the thermogravimetric analysis (TGA; Figure S10, Supporting Information).

**Figure 2:**
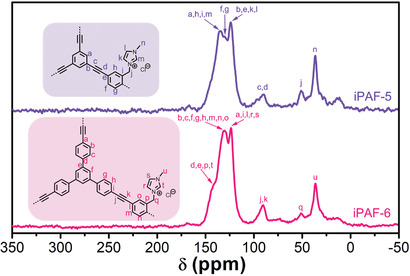
Solid‐state ^13^C CP/MAS nuclear magnetic resonance (NMR) spectra of iPAF‐5 and iPAF‐6.

To demonstrate the porous nature of prepared PAFs, nitrogen adsorption−desorption isotherms were performed at 77 K (Figure S11a, Supporting Information). The BET surface areas are measured as 208 and 148 m^2^ g^−1^ for iPAF‐5 and iPAF‐6, respectively. The obtained surface areas are lower than common PAF materials because of the introduction of imidazolium groups onto the frameworks. The pore size distributions were calculated by nonlocal density functional theory (NLDFT) (Figure S11b, Supporting Information). Both micropores and mesopores exist in iPAF‐5 and iPAF‐6 samples. However, iPAF‐6 shows more mesopores distributions and larger mesopore volume (Table S2, Supporting Information), which are beneficial to the diffusion of guest molecules into the PAFs and increase the accessible adsorption sites. The porous nature results are also in accordance with the previous study, which indicates that increasing the monomer structure length tunes the pore sizes to larger ones but decreases the surface areas.^[^ ^16^ ^]^

After their physiochemical characterization, the bilirubin adsorption performances were examined. To simulate the pH environment of human blood, phosphate buffered saline (PBS, pH = 7.4) was used to prepare bilirubin adsorption solution. To further demonstrate the charge and pore size influence on the bilirubin adsorption, we also synthesized uncharged PAFs (PAF‐uc, from 1,4‐dibromobenzene (DBB) and TEPB) and linear cationic polymer without porosity (iLP, from DBMIIB and 1,4‐diethynylbenzene (DTB)) (Figure S11, Table S2, and Table S3, Supporting Information) for comparison. PAF‐uc has larger surface area and larger mesopore volume than iPAF‐6. However, iLP has almost no pores. First, the changes in bilirubin concentration against adsorption time by different adsorbents were investigated (**Figure** **3**a). Commercial adsorbents such as AC and anion exchange resin (AER) were also included in this experiment and their product details are shown in the Supporting Information. Compared with other adsorbents, iPAF‐5 and iPAF‐6 show faster concentration decreases and higher removal efficiencies. In addition, the bilirubin can be adsorbed to normal concentration in just 5 min by iPAF‐6. The final residual concentrations are 1.23 and 0.18 mg L^−1^ for iPAF‐5 and iPAF‐6, respectively. To evaluate the affinity of the adsorbents quantitatively, the distribution coefficient (*K*_d_) was employed. Normally, *K*_d_ value above 1.0 × 10^5^ mL g^−1^ is considered as excellent.^[^ ^8a^ ^]^ As shown in Figure 3b, iPAF‐5 and iPAF‐6 have much higher *K*_d_ values than other adsorbents in this work and iPAF‐6 shows the highest *K*_d_ value of 1.04 × 10^6^ mL g^−1^ demonstrating its high affinity toward bilirubin. The bilirubin uptake by the adsorbents during the adsorption processes was calculated and the data were studied by the pseudo‐second‐order kinetic model (Figure S12, Supporting Information). All the adsorption processes fit quite well with this model (correlation coefficient *R* ^2^ > 0.9950). The highest rate constant *K*_2_ for iPAF‐6 (2.72 × 10^−2^ g mg^−1^ min^−1^, one order of magnitude larger than that of other tested adsorbents; Table S4, Supporting Information) suggests that iPAF‐6 has the fastest adsorption kinetics, and it can reach the adsorption equilibrium within 25 min. Next, the adsorption isotherms of PAF‐uc, iLP, iPAF‐5, and iPAF‐6 were conducted to further understand the adsorption properties (Figure 3c and Figure S13, Supporting Information). Two widely used isotherm models, namely Langmuir model and Freundlich model, were first employed to fit the isotherm data (Figure S14, Supporting Information). According to the correlation coefficient *R* ^2^ and fitting curves, the isotherm data can be all better described by Langmuir model (Table S5, Supporting Information). The maximum adsorption capacities from Langmuir model for iLP, iPAF‐5, and iPAF‐6 are 556, 862, and 1296 mg g^−1^. However, these values are high for these adsorbents with surface areas below 300 m^2^ g^−1^, indicating that monolayer adsorption of Langmuir model may not be the most suitable one. Thus, it is therefore thought worthwhile to analyze the isotherm data with Zhu and Gu isotherm model (Figure 3c and Figure S15, Supporting Information).^[^ ^17^ ^]^ The fitting results demonstrate that the adsorption by iLP, iPAF‐5, and iPAF‐6 are more suitable to be explained by Zhu and Gu model with higher *R* ^2^ values (Table S6, Supporting Information), indicating these adsorption processes involve two steps: the first step is the adsorption interactions between bilirubin and the adsorption sites in adsorbents, whereas the second step is the self‐aggregation of bilirubin molecules on the surfaces of adsorbents because of hydrogen bonding and hydrophobic interaction.^[^ ^17^ ^]^ Remarkably, the maximum adsorption capacity (*q*_m_) of iPAF‐6 from Zhu and Gu model is 1249 mg g^−1^, which is also the highest among the tested samples. When both maximum adsorption capacity from isotherm data and equilibrium adsorption time from kinetic data are considered, the adsorption performance of iPAF‐6 is the best one among the reported novel bilirubin adsorbents (Figure 3d). Based on the high *K*_d_ value, fast adsorption kinetics and large maximum adsorption capacity, iPAF‐6 clearly outperforms PAF‐uc, iLP, and iPAF‐5. Compared with nonporous iLP, iPAF‐5 and iPAF‐6 have better adsorption performances though the content of imidazolium groups for iLP is similar to that for iPAF‐5, which indicate that the porosity of PAFs can expose more available adsorption sites for bilirubin. Compared with uncharged PAF‐uc, iPAF‐5 and iPAF‐6 also have better adsorption performances suggesting that positively charged groups are important for bilirubin adsorption. To further demonstrate the intrinsic driving force for bilirubin adsorption by iPAF‐5 and iPAF‐6, we performed the density functional theory (DFT) studies to analyze the interaction between the repeating unit as a fragment of PAFs and bilirubin (Figure 3e). The binding energies are in the order of iPAF‐6···bilirubin > iPAF‐5···bilirubin > PAF‐uc···bilirubin, which agrees with the experimental adsorption capacity results. Typical *π*−*π* interaction can be observed between benzene rings from PAFs and pyrrole rings. In addition, the ion exchange process is driven by the electrostatic interaction (between positively charged imidazolium group and carboxyl groups from bilirubin) exist in iPAF‐5···bilirubin and iPAF‐6···bilirubin. The ion exchange process can be also verified by EDS elemental mapping of iPAF‐6 after bilirubin adsorption (Figure S16, Supporting Information). Compared with PAF‐uc···bilirubin showing only *π*−*π* interaction, the binding energies of iPAF‐5···bilirubin and iPAF‐6···bilirubin have sharp increases from −15.6 kcal mol^−1^ to −52.5 and −76.4 kcal mol^−1^, respectively, suggesting that the electrostatic interaction derived from imidazolium groups is the dominant driving force for bilirubin adsorption by iPAF‐5 and iPAF‐6. Between iPAF‐5 and iPAF‐6, iPAF‐6 shows a higher binding energy. The longer structure length of TEPB for iPAF‐6 can lead to a larger pore size which not only improves the mass transfer of bilirubin into the internal space of PAFs but also makes bilirubin have a suitable molecular configuration with a stronger electrostatic interaction. These results suggest that the superior adsorption performance of iPAF‐6 should be attributed to its multiple adsorption sites and larger pore size.

**Figure 3:**
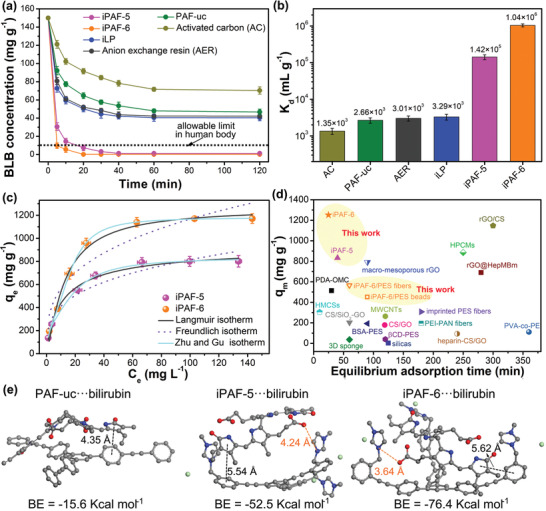
a) Bilirubin (BLB) removal versus contact time by different tested adsorbents (initial bilirubin concentration *C*_0_ = 150 mg L^−1^, adsorbent dosage m/V = 0.8 g L^−1^). b) *K*_d_ values of the tested adsorbents. c) Bilirubin adsorption isotherms for iPAF‐5 and iPAF‐6 (*C*_0_ = 35−500 mg L^−1^, m/V = 0.25 g L^−1^). d) Comparison of bilirubin maximum adsorption capacity (*q*_m_) and equilibrium adsorption time for our prepared adsorbents with other reported novel bilirubin adsorbents, PDA‐OMC,^[^ ^4a^ ^]^ HMCSs,^[^ ^18^ ^]^ CS/SiO_2_‐GO,^[^ ^6e^ ^]^ BSA‐PES,^[^ ^19^ ^]^ 3D sponge,^[^ ^20^ ^]^ MWCNTs,^[^ ^21^ ^]^ CS/GO,^[^ ^4b^ ^]^ *β*‐CD‐PES,^[^ ^6a^ ^]^ silicas,^[^ ^6d^ ^]^ macromesoporous rGO,^[^ ^6c^ ^]^ HPCMs,^[^ ^22^ ^]^ imprinted PES fibers,^[^ ^23^ ^]^ PEI‐PAN fibers,^[^ ^6b^ ^]^ heparin‐CS/GO,^[^ ^24^ ^]^ rGO/CS,^[^ ^25^ ^]^ rGO@HepMBm,^[^ ^5b^ ^]^ and PVA‐co‐PE.^[^ ^26^ ^]^ e) Density functional theory (DFT) optimized adsorption complexes of bilirubin with PAF‐uc, iPAF, and iPAF‐6, and their calculated binding energies.

With such promising results for iPAF‐6 to remove bilirubin, we further illustrated its practical application potential through adsorption selectivity against bovine serum albumin (BSA), recyclability, biocompatibility, and simulated hemoperfusion experiments. An ideal bilirubin adsorbent should also have the ability to extract bilirubin from albumin‐rich solution.^[^ ^6^, ^24^ ^]^ Because bilirubin is an albumin‐bound toxin in the blood and lots of albumin exist in the plasma or blood which inhibits the removal of bilirubin during the hemoperfusion process. In this work, BSA replaces human serum albumin (HSA) as the model albumin to study the influence of albumin on bilirubin adsorption because of their similar molecular structure and nearly equal bilirubin binding ability.^[^ ^6b^ ^]^ As shown in **Figure** **4**a, when the concentration of BSA reaches as high as 50 g L^−1^, the removal efficiency is still above 96% and the adsorption amount is 192 mg g^−1^, which is also higher than other bilirubin adsorbents under the coexistence of albumin (Table S7, Supporting Information). The BSA removal efficiency during the adsorption processes was also investigated, which is less than 0.2% even at the high concentration of BSA (50 g L^−1^), suggesting that iPAF‐6 cannot cause obvious albumin loss (Figure S17, Supporting Information). The low removal efficiency toward albumin by iPAF‐6 is due to that the size of albumin is obviously larger than the pore size of iPAF‐6. The result indicates that iPAF‐6 has a good adsorption selectivity toward bilirubin against albumin interference. The regeneration ability of the adsorbent is also important for reducing the overall costs. The saturated bilirubin‐adsorbed iPAF‐6 was immersed into the eluent (20 mL mixture solution of 5 m NaCl and 1 m NaOH) for 1 h to conduct the desorption, followed by H_2_O and ethanol washing. After five adsorption‐regeneration cycles, the removal efficiency still remains at 94% (Figure 4b), indicating its good reusability and stability.

**Figure 4:**
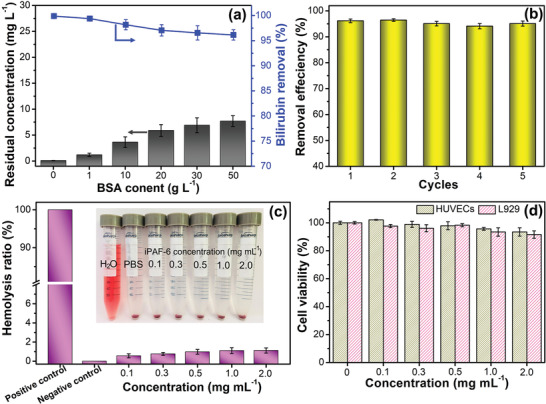
a) Residual concentration and removal efficiency of bilirubin by iPAF‐6 in the presence of varying concentrations of bovine serum albumin (BSA; *C*_0_ = 200 mg L^−1^, m/V = 0.1 g L^−1^). b) Reusability tests of iPAF‐6 toward bilirubin in the presence of 50 g L^−1^ BSA (*C*_0_ = 200 mg L^−1^, m/V = 0.1 g L^−1^). c) Hemolysis rate results for control groups and different concentrations of iPAF‐6 (inset is digital photo of red blood cells incubated with different samples). d) The cell viability in L929 fibroblast cells and HUVECs at varying concentration of iPAF‐6 by CCK‐8 assay (the control group was the fresh culture medium without any samples).

As a hemoperfusion adsorbent which has a direct contact with blood or plasma, good biocompatibility is necessary. To demonstrate this property, the hemolysis ratio, coagulation time, platelet activation, complement activation, and cytotoxicity of iPAF‐6 were tested. During the hemolysis assay, hemolysis will lead to the hemoglobin release into the solution and the resulting solution will become red visually. The lower hemolysis ratio means the better blood compatibility.^[^ ^4a^ ^]^ As shown in Figure 4c, in contrast to negative control, no visible hemolysis phenomena can be observed for iPAF‐6 groups even at the high dosage of 2 mg mL^−1^. The hemolysis ratio of iPAF‐6 at this dosage is 1.1%, which is far below the standard established by the American Society of Testing Materials (ASTM) (5%).^[^ ^5b^ ^]^ Coagulation time tests including activated partial thromboplastintime (APTT), thrombin time (TT), and prothrombin time (PT) were performed to study the coagulant property (Figure S18, Supporting Information). The APTT and TT can assess the antithrombogenicity of the samples in vitro and PT can confirm the exogenous coagulation ability. Compared with the control group of platelet‐poor plasma, the values of APTT, TT, and PT for iPAF‐6 in the tested dosage (1.0−20.0 mg) show no significant decreases. The small differences suggest that iPAF‐6 cannot induce any obvious coagulation. In addition, the blood compatibility of iPAF‐6 in the whole blood medium including whole blood clotting times, platelet activation, and complement activation was further investigated. Compared with control group, the whole blood clotting time for iPAF‐6 at the high concentration (2 mg mL^−1^) has no significant reduction (Figure S19, Supporting Information), suggesting that iPAF‐6 shows little whole blood coagulation effect. We used PF4 concentration level to evaluate the platelet activation for iPAF‐6. The PF4 concentration shows almost no increase for iPAF‐6 at the tested concentrations compared with control group (Figure S20, Supporting Information), suggesting that iPAF‐6 cannot induce the platelet activation. C3a and C5a are the activation products of complement system, which are used to evaluate complement activation. There is no obvious difference between control sample and iPAF‐6 groups for C3a and C5a (Figure S21, Supporting Information), which indicates that no inflammation response would be activated when iPAF‐6 contacts with the blood. The results reveal that iPAF‐6 has good blood compatibility. The cytotoxicity of iPAF‐6 over in L929 fibroblast cells and human umbilical vein endothelial cells (HUVECs) were investigated. As shown in Figure 4d, the cell viabilities are over 90% at all concentrations of the iPAF‐6 suspension. Moreover, the cells after culturing for 3 days were stained with TRITC Phalloidin and DAPI and were observed by the laser confocal fluorescence microscope. The culture with iPAF‐6 (2.0 mg mL^−1^) does not change the cellular morphology when compared to the control groups (Figure S22, Supporting Information). Cell viabilities and fluorescence images can confirm the nontoxicity of iPAF‐6. The biocompatibility tests conclude that iPAF‐6 has the potential in a clinical application.

Clinical hemoperfusion involves the direct flowing of human blood over an adsorbent system. The dynamic adsorption process is needed to investigate the practical application ability. To simulate real hemoperfusion equipment for bilirubin removal, a laboratory‐made continuous flow system containing the adsorption column connected with peristaltic pump was constructed (Figure S23, Supporting Information). PBS composed of 200 mg L^−1^ bilirubin, 50 g L^−1^ BSA, and 9 g L^−1^ NaCl was prepared to simulate hyperbilirubinemia patients’ plasma. Previous studies have demonstrated that the powder form of porous materials restricted their practical usage as the adsorption columns due to the clogging, aggregation, and difficult recycle.^[^ ^27^ ^]^ In this work, iPAF‐6/polymer composite beads and fibers were prepared to address these issues (**Figure** **5**a,b). Polyethersulfone (PES) was selected as the polymer matrix to load and disperse iPAF‐6. Because PES has good stability, mechanical property and biocompatibility, and pure PES fibers and beads have been used for the bilirubin removal.^[^ ^23^, ^28^ ^]^ The SEM images demonstrate that an irregular spongy‐like macropore structures are in the inner part of iPAF‐6/PES bead and iPAF‐6 particles are loaded into the spongy‐like pores (Figure 5a and Figure S24, Supporting Information). iPAF‐6/PES fibers show a hierarchical pore structure among the fibers due to the fiber accumulation and iPAF‐6 particles are inside the fibers or on the surfaces of the fibers (Figure 5b). The macropore structures in the composites are beneficial to the contact between iPAF‐6 and bilirubin. The static adsorption of the bead/fiber materials was first studied. Compared with pure PES fibers and beads, iPAF‐6/PES fibers and beads have faster bilirubin removal rates and higher adsorption capacities (Figure S25, Figure S26, and Table S8−S10, Supporting Information). It can be seen that the adsorption capacity of the composites comes mainly from iPAF‐6 and iPAF‐6 plays a decisive role. The equilibrium adsorption time for iPAF‐6/PES beads and iPAF‐6/PES fibers are 90 and 60 min, respectively. The maximum adsorption capacities for iPAF‐6/PES beads and iPAF‐6/PES fibers from adsorption isotherm model are 448.76 and 562.23 mg g^−1^, respectively. Although the adsorption performance of the iPAF‐6/PES composites are not as good as pure iPAF‐6 owning to the low content of iPAF‐6 in the composites (30 wt%), they are still better than most of the reported novel bilirubin adsorbents (Figure 3d). We will improve our preparation technique to increase the content of iPAF‐6 in the composites in the future study. Then, the dynamic hemoperfusion adsorption was carried out. As show in Figure 5c, iPAF‐6/PES beads and fibers have faster removal rates and higher removal efficiencies than pure PES beads (or fibers) and commercial adsorbents (AC and AER) because of the good adsorption performance of iPAF‐6. The bilirubin can be adsorbed to normal concentration within 1 h by iPAF‐6/PES fibers and iPAF‐6/PES beads need 2 h. The final concentrations of bilirubin after the dynamic adsorption processes treated with iPAF‐6/PES beads and iPAF‐6/PES fibers are 5.31 and 1.02 mg L^−1^, respectively. The better performance of iPAF‐6/PES fibers in comparison to iPAF‐6/PES beads is attributed to their small fiber diameters and fibrous structures, which can make iPAF‐6 a more fully contact with bilirubin molecules. The iPAF‐6 leakage from the composite beads and fibers after the hemoperfusion adsorption was evaluated by the weight loss. The weight losses for iPAF‐6/PES beads and iPAF‐6/PES fibers are only 0.16% and 0.11%, respectively, indicating that iPAF‐6 can be steadily loaded into the composites.

**Figure 5:**
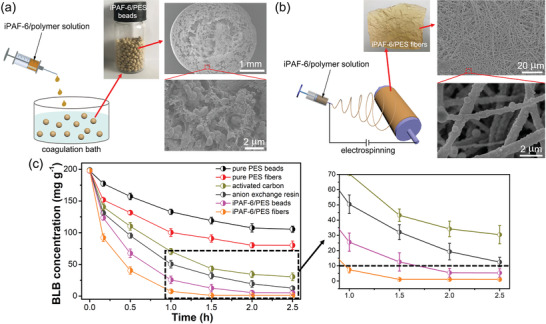
Schematic illustration for the fabrication of a) iPAF‐6/PES beads and b) fibers (insets are their digital photographs and SEM images). c) Bilirubin (BLB) removal from simulated hyperbilirubinemia patients’ plasma by different adsorbents under the hemoperfusion process.

In conclusion, for the first time, we have demonstrated the outstanding performance of PAF‐based materials as hemoperfusion adsorbents for the removal of bilirubin. Through targeted function design, cationic iPAF‐6 with lager pore sizes show a remarkable adsorption affinity toward bilirubin because of its abundant binding sites (providing electrostatic interactions and *π*−*π* interactions) and sufficient space for fast diffusion. As a result, iPAF‐6 achieves fast adsorption kinetics within 25 min and ultrahigh adsorption capacity of 1249 mg g^−1^. Due to its high affinity and constitutive structure, iPAF‐6 can also remove ≈96% of the bilirubin under the presence of 50 g L^−1^ BSA with an adsorption amount of 192 mg g^−1^. The superior adsorption performance of iPAF‐6 outperforms most reported bilirubin adsorbents in terms of adsorption capacity, kinetics, and selectivity. Moreover, the biocompatible building units make iPAF‐6 be nontoxic and hemocompatible. More importantly, iPAF‐6 powders can be processed into bead or fiber forms with the assistance of PES that makes iPAF‐6 be practically favorable for the dynamic hemoperfusion. iPAF‐6/PES composite beads or fibers show rapid and efficient bilirubin removal in the continuous‐flow system, which surpass commercial ACs and AERs. This work gives new insights into the design of bilirubin adsorbents and demonstrates the great potential of PAF‐based adsorbents in hemoperfusion applications.

## Conflict of Interest

The authors declare no Conflict of interest.

## Supporting information

Supporting Information
