# Supplementary material for: Porous Aromatic Framework with Tailored Binding Sites and Pore Sizes as a High‐Performance Hemoperfusion Adsorbent for Bilirubin Removal
Source: Adv Sci (Weinh). 2020 Oct 25;7(23):2001899. doi: 10.1002/advs.202001899 (PMC7709998; doi:10.1002/advs.202001899)
Supplement: Supplementary file 1 — Supporting Information [file ADVS-7-2001899-s001.pdf]

© 2020 Wiley-VCH GmbH

Supporting Information

for *Adv. Sci.,* DOI: 10.1002/advs.202001899

Porous aromatic framework with tailored binding sites and pore sizes as a high-performance hemoperfusion adsorbent for bilirubin removal

Rui Zhao, Tingting Ma, Fengchao Cui, Yuyang Tian, and Guangshan Zhu**

© 2020 Wiley-VCH GmbH

Supporting Information

Porous aromatic framework with tailored binding sites and pore sizes as a high-performance hemoperfusion adsorbent for bilirubin removal

Rui Zhao, Tingting Ma, Fengchao Cui, Yuyang Tian and Guangshan Zhu**

Faculty of Chemistry, Northeast Normal University, Changchun, 130024, China

Experimental Section

Materials

All reagents and anhydrous solvents of analytical purity were commercially available and used as received unless otherwise indicated. Poly(ether sulfones) (PES, E1010) were supplied by BASF chemical company (Germany). Granular activated carbon with the specific surface area of 500~1000 m^2 g^{-1} was purchased from Aladdin. Anion exchange resin (D201, functional group: quaternary ammonium group, ion exchange capacity: ≥ 3.70 mmol g^{-1}) was purchased from Tianjin BSF resin technology Co. Ltd. Bilirubin was obtained from Aladdin and stored at -20 °C. 1,3,5-tris(4-ethynylphenyl)benzene was purchased from Yanshen Technology Co., Ltd (China).

Material synthesis

Synthesis of 3-(2,5-dibromobenzyl)-1-methyl-1H-imidazol-3-ium bromide.

The preparation of linear cationic building monomer (3-(2,5-dibromobenzyl)-1-methyl-1H-imidazol-3-ium bromide, DBMIIB) complied with the following route.

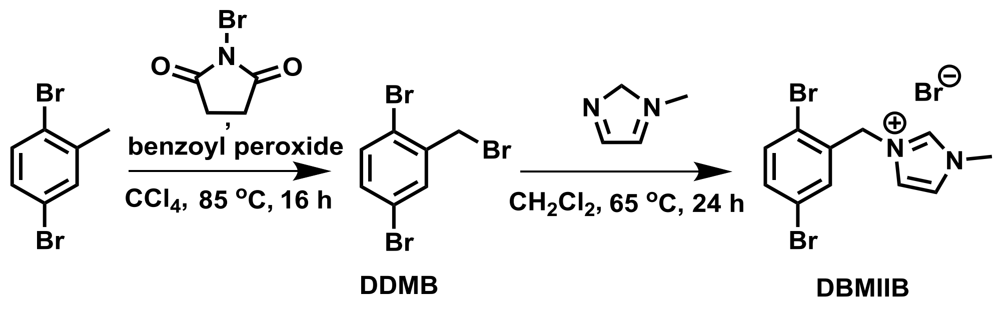

Synthesis of 1,4-dibromo-2-(bromomethyl)benzene (DDMB): The synthesis of DDMB followed a modified procedure from reference.[1] 1,4-dibromo-2-ethylbenzene (1.390 g, 5.27 mmol) was dissolved into 100 mL anhydrous CCl_4 under the stirring.

This mixture was degassed with a stream of nitrogen for 15 minutes. Then, N-bromosuccinimide (1.780 g, 10.00 mmol) and benzoyl peroxide (0.0485 g, 0.20 mmol) were added under N_2 protection. The mixture was heated at 85 °C under N_2 for 16 h. After being cooled, the resulting precipitate was filtered to obtain the crude compound which was purified by column chromatography to afford the title compound as a white solid (0.745 g, 2.26 mmol, 43% yield). ^{1}H NMR (600 MHz, $CDCl_3$): δ 7.53 (1H, Ar-*H*), 7.37 (1H, Ar-*H*), 7.23 (1H, Ar-*H*), 4.46 (2H, C*H*$_2$Br).

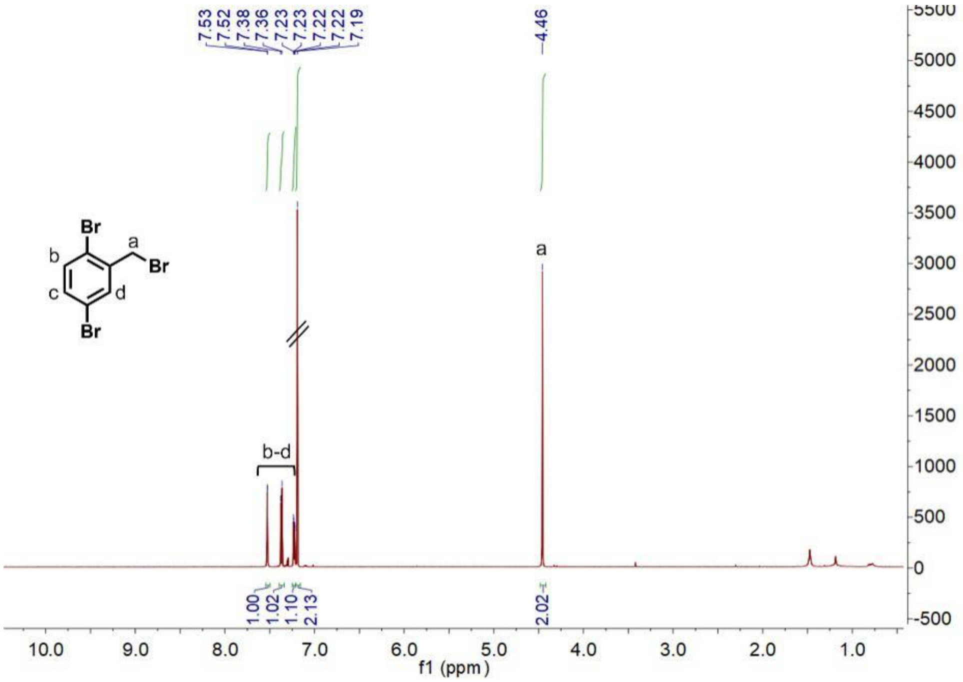

Figure S1. ^{1}H NMR of DDMB using the solvent of $CDCl_3$.

Synthesis of DBMIIB: The synthesis of DBMIIB followed a modified procedure from reference.[1] A mixture of DDMB (0.631 g, 1.92 mmol), N-methylimidazole (0.204 g, 2.48 mmol), and CH_2Cl_2 (50 mL) was heated at 65 °C under N_2 atmosphere for 24 h. After being cooled, the resulting precipitate was filtered, washed with ethyl acetate, and dried in vacuo to afford the product as a white solid (0.757 g, 1.84 mmol, 96% yield). ^{1}H NMR (600 MHz, d_6-DMSO): δ 9.17 (1H, Imidazol-*H*), 7.80 (1H, Imidazol-*H*), 7.77 (1H, Imidazol-*H*), 7.70 (1H, Ar-*H*), 7.65 (1H, Ar-*H*), 7.60 (1H, Ar-*H*), 5.50 (2H, C*H*$_2$Br), 3.88 (3H, C*H*$_3$).

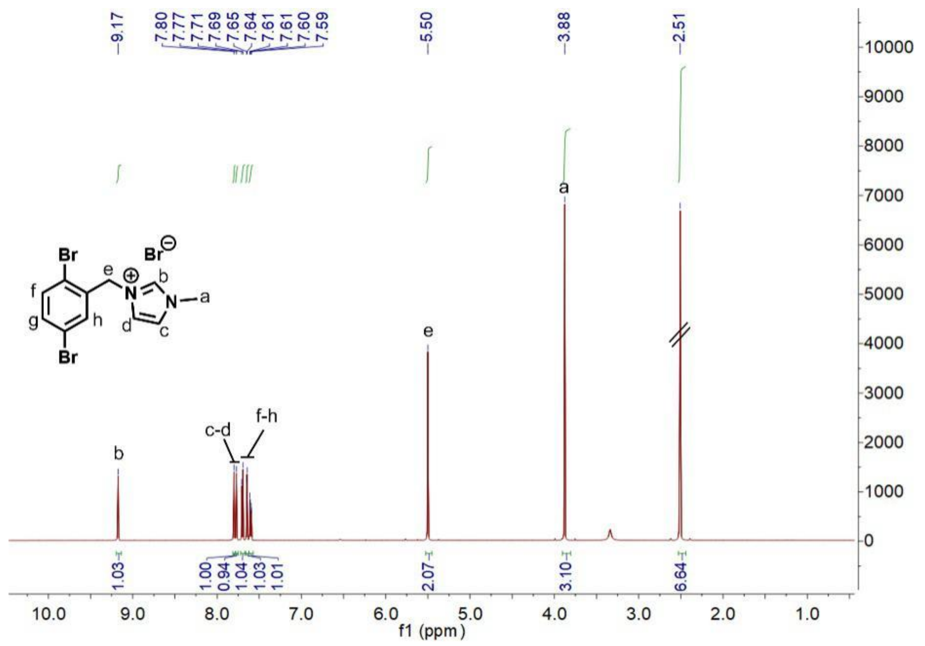

Figure S2. ^{1}H NMR of DBMIIB using the solvent of d_6-DMSO.

Synthesis of iPAF-5. DBMIIB (0.370 g, 0.90 mmol) and 1,3,5-triethynylbenzene (0.090 g, 0.60 mmol) were added to a mixture solution of tetrakis(triphenylphosphine)palladium(0) (0.150 g, 0.21 mmol), CuI (0.050 g, 0.26 mmol) in N,N-dimethylformamide (DMF) (15 mL) and triethylamine (15 mL). This mixture was degassed with a stream of nitrogen. The reaction mixture was refluxed at 90 °C for 12 h and 120 °C for 48 h under nitrogen protection. After cooling to room temperature, the solid products were filtered and washed with chloroform, acetonitrile, DMF and methanol. Then, the products were added into 1 M HCl solution (100 mL) overnight to conduct the ion-exchange process from Br^- to Cl^-. The products were then purified by Soxhlet extraction with methanol for 48 h and subsequently dried under vacuum for 24 h at 80 °C to yield iPAF-5 as a brown powder (0.186 g, 67.7% yields).

Synthesis of iPAF-6. DBMIIB (0.370 g, 0.90 mmol) and 1,3,5-tris(4-ethynylphenyl)benzene (0.227 g, 0.60 mmol) were added to a mixture solution of tetrakis(triphenylphosphine)palladium(0) (0.150 g, 0.21 mmol), CuI

(0.050 g, 0.26 mmol) in DMF (15 mL) and triethylamine (15 mL). This mixture was degassed with a stream of nitrogen. The reaction mixture was refluxed at 90 °C for 12 h and 120 °C for 48 h under nitrogen protection. After cooling to room temperature, the solid products were filtered and washed with chloroform, acetonitrile, DMF and methanol. Then, the products were added into 1 M HCl solution (100 mL) overnight to conduct the ion-exchange process from Br^{-} to Cl^{-}. The products were then purified by Soxhlet extraction with methanol for 48 h and subsequently dried under vacuum for 24 h at 80 °C to yield iPAF-6 as a brown powder (0.263 g, 63.9% yields).

Synthesis of PAF-uc. 1,4-dibromobenzene (DBB) (0.212 g, 0.90 mmol) and 1,3,5-tris(4-ethynylphenyl)benzene (TEPB) (0.227 g, 0.60 mmol) were added to a mixture solution of tetrakis(triphenylphosphine)palladium(0) (0.150 g, 0.21 mmol), CuI (0.050 g, 0.26 mmol) in DMF (15 mL) and triethylamine (15 mL). This mixture was degassed with a stream of nitrogen. The reaction mixture was refluxed at 90 °C for 12 h and 120 °C for 48 h under nitrogen protection. After cooling to room temperature, the solid products were filtered and washed with chloroform, acetonitrile, DMF and methanol. The products were then purified by Soxhlet extraction with methanol for 48 h and subsequently dried under vacuum for 24 h at 80 °C to yield PAF-uc (0.205 g, 69.8% yields).

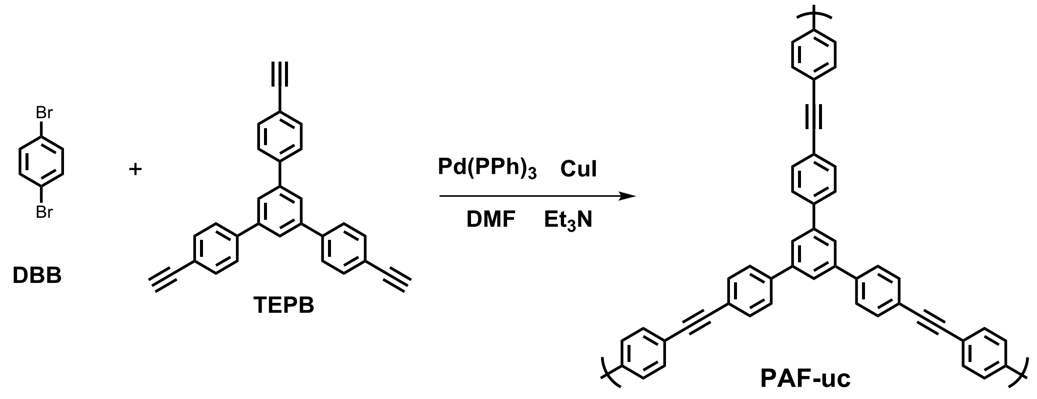

Synthesis of iLP. DBMIIB (0.370 g, 0.90 mmol) and 1,4-diethynylbenzene (DTB) (0.114 g, 0.90 mmol) were added to a mixture solution of tetrakis(triphenylphosphine)palladium(0) (0.150 g, 0.21 mmol), CuI (0.050 g, 0.26

mmol) in DMF (15 mL) and triethylamine (15 mL). This mixture was degassed with a stream of nitrogen. The reaction mixture was refluxed at 90 °C for 12 h and 120 °C for 48 h under nitrogen protection. After cooling to room temperature, the solid products were filtered and washed with chloroform, acetonitrile, DMF and methanol. Then, the products were added into 1 M HCl solution (100 mL) overnight to conduct the ion-exchange process from Br^- to Cl^-. Then, the products were dried under vacuum for 24 h at 80 °C to yield iLP (0.185 g, 61.6% yields).

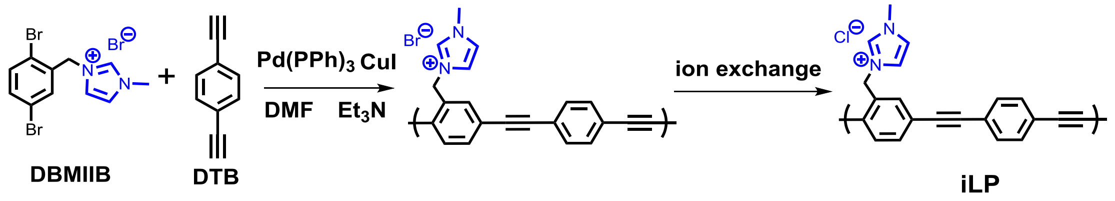

Preparation of iPAF-6/polymer composites. To improve the practicability of iPAF-6 powders as adsorption columns, iPAF-6/polyethersulfone (PES) composite beads and fibers were fabricated. 0.75 g iPAF-6 powder was dispersed in 7.5 g DMF under the sonication for 4 h. Then, 1.75 g PES was dissolved in the solution under continuous mechanical stirring, followed by adding 1.00 g polyethylene glycol (PEG, M_w = 800). After they completely dissolved, the composite solution was obtained. For iPAF-6/PES bead preparation, the composite solution was added drop-by-drop into the coagulation bath ($V_{water}/V_{ethanol}$ = 1:1) to form the beads. For iPAF-6/PES fiber preparation, the composite solution was loaded into a 10 mL syringe to conduct the electrospinning process. 18 kV was applied between the cathode and anode at a distance of 15 cm with a flow rate of 1.0 mL h^{-1}. The iPAF-6/PES fibers were collected using a metallic rotating roller drum. The obtained beads and fibers were washed with water and methanol, and dried under vacuum at 120 °C for 12 h before use. Pure PES beads and fibers without iPAF-6 doping were also prepared with the same procedures.

Adsorption Experiments

To obtain a homogeneous bilirubin solution, a certain amount of bilirubin was initially

dissolved in a small volume of DMSO and 0.1 M Na_2CO_3 solution, and then diluted with phosphate buffer saline (PBS, pH = 7.4) to get the desired concentration. In order to avoid the degradation of bilirubin, solution preparation and adsorption experiments were conducted under dark conditions. All the adsorption experiments were performed in a thermostat water bath at 37 °C.

Bilirubin adsorption kinetics. 40 mg of the adsorbents were added into 50 mL bilirubin solution (150 mg L^{-1}). The mixture was shaken for 2 h at 37 °C. At appropriate time intervals, aliquots (1 mL) were taken from the mixture, and the aliquots were filtrated through a syringe filter (0.45 μm membrane filter). Then, the remaining bilirubin concentration was determined. The adsorption capacity (q) and removal efficiency (R%) of bilirubin by the adsorbent was calculated on the basis of the following equation:

$$\mathrm{q}\ (mg\ g^{-1}) = \frac{(\mathrm{C_0} - \mathrm{C_e})\mathrm{V}}{\mathrm{W}}$$

$$\mathrm{R}\ (\%) = \frac{(\mathrm{C_0} - \mathrm{C_e})}{C_0} \times 100\%$$

where C_0 and C_e (mg L^{-1}) are the initial and the equilibrium concentration of bilirubin in the solution, respectively. V (L) is the volume of the solution, and W (g) is the mass of the adsorbent. The kinetics data were further analyzed by the pseudo-second-order kinetic model.[2] Its linear equation is listed as follows:

$$\frac{\mathrm{t}}{\mathrm{q_t}} = \frac{1}{\mathrm{k_2 q_e^2}} + \frac{\mathrm{t}}{\mathrm{q_e}}$$

where q_t and q_e (mg g^{-1}) are the adsorption capacity at time t and equilibrium time, respectively. k_2 (g mg^{-1} min^{-1}) is the pseudo-second order model rate constant.

K_d value calculation. 40 mg of the adsorbents were added into 50 mL bilirubin solution (150 mg L^{-1}). After the adsorption for 2 h, the adsorbent was separated by syringe filter (0.45 μm membrane filter). The bilirubin concentrations in the resulting solutions were analyzed and the K_d values were calculated as:

$$K_d = (\frac{C_0 - C_e}{C_e}) \times \frac{V}{m}$$

where V is the volume of the adsorption solution (mL), m is the weight of adsorbent

(g), C_0 and C_e the initial and equilibrium concentrations, respectively.

Bilirubin adsorption isotherms. 5 mg of the adsorbents were added into 20 mL bilirubin solutions with concentrations ranging from 35 to 500 mg L^{-1}. After adsorption equilibrium, the solution was filtered through a 0.45 μm syringe filter. The remaining bilirubin concentrations were analyzed and the adsorption capacities were calculated. The isotherm data were analyzed by two isotherm models, namely Langmuir and Freundlich,[3] whose linear equations are expressed as follows:

Langmuir isotherm (homogeneous and monolayer adsorption):

$$\frac{C_e}{q_e} = \frac{C_e}{q_m} + \frac{1}{bq_m}$$

Freundlich isotherm (heterogeneous and multilayer adsorption):

$$\log q_e = \log K_F + \frac{1}{n}\log C_e$$

where q_e is the equilibrium adsorption capacity (mg g^{-1}), C_e is the equilibrium concentration (mg L^{-1}), and q_m and b are Langmuir constants related to maximum adsorption capacity and binding energy, respectively; K_F and n are empirical constants that indicate the Freundlich constant and heterogeneity factor, respectively.

The isotherm data were also analyzed by Zhu and Gu isotherm model (which involves two steps: the first step includes the interaction between the adsorbate molecule and the adsorbent, while the second step involves the creation of hemimicelles between adsorbed molecules through intermolecular interaction on the surface of the adsorbents) with the following equation:[4]

$$q_e = q_m \frac{K_1 C_e(\frac{1}{m} + K_2 C_e^{m-1})}{1 + K_1 C_e(1 + K_2 C_e^{m-1})}$$

where K_1 and K_2 are the equilibrium adsorption constants, and m is the average aggregation number of the hemimicelles.

The effect of albumin on bilirubin adsorption by iPAF-6. 5 mg of iPAF-6 was added into 5 mL bilirubin solution (200 mg L^{-1}) with different bovine serum albumin

(BSA) concentration (0-50 g L^{-1}). Different content of BSA was directly added to the bilirubin solution and stirred to dissolve completely before use. After adsorption equilibrium, the solution was filtered through a 0.45 μm syringe filter. The remaining bilirubin concentrations were analyzed and the removal efficiencies were calculated. After adsorption equilibrium, the removal efficiencies of iPAF-6 towards BSA were also calculated. The BSA concentration was determined using the BCA Protein Assay Kit.

Reusability tests for iPAF-6. For the regenerative experiment, 5 mg adsorbent was added into 5 mL albumin-bonded bilirubin solution (bilirubin concentration: 200 mg L^{-1}, BSA concentration: 50 g L^{-1}). The mixture was shaken for 2 h at 37 °C. After adsorption equilibrium, the adsorbent was filtered and was regenerated by 20 mL eluent (containing 5 M NaCl and 1 M NaOH). After washing thoroughly with deionized water and ethanol to neutral pH, the adsorbent was reused in adsorption experiments. The experimental protocol was repeated five times.

Simulated hemoperfusion experiment. To simulate the clinical hemoperfusion device, a peristaltic pump with a glass column (100 mm in length and 16 mm in diameter) was applied to study the batch adsorption experiment. The column was packed with 10 mL of different adsorbents (activated carbon: ~6.7 g, anion exchange resin: ~5.1 g, beads: ~4.2 g, fibers: ~3.6 g) and the adsorbents were equilibrated with deionized water prior to packing. PBS with 200 mg L^{-1} bilirubin, 50 g L^{-1} BSA, and 9 g L^{-1} NaCl was prepared to simulate hyperbilirubinemia patients' plasma. 1000 mL simulated hyperbilirubinemia patients' plasma was pumped down-flow through the adsorbent column circularly by the peristaltic pump at a constant flow rate of 150 mL min^{-1}. Solution and column were equipped with a water bath to maintain a constant temperature at 37 °C. At appropriate time intervals, the bilirubin concentration in the solution was analyzed. After the hemoperfusion adsorption, composite beads and fibers were desorption by the above-mentioned eluent. After the desorption the

composites were washed with deionized water and ethanol, and then the composites were placed in a vacuum oven to dry until constant weight. The iPAF-6 leakage from the composite beads and fibers were evaluated by the weight loss after the hemoperfusion based on the following equation:

$$Weight\ loss\ (\%) = \frac{W_0 - W_i}{W_0} \times 100\%$$

where W_0 and W_i are the weights of the initial samples and the samples after drying in a vacuum oven, respectively.

Computational method

To understand the intrinsic driving force for bilirubin adsorption by iPAF-5 and iPAF-6, we evaluated the interaction energies between the repeating unit as a fragment of PAFs and bilirubin using quantum chemical calculations. The 3D structures of iPAF-5, iPAF-6, and PAF-uc were built with GaussView5.0.8 and bilirubin 3D structure was download from pubchem website. All calculations were carried out using the density functional theory (DFT) with the B3LYP functiona[5,6] as implemented in the Gaussian 09 program.[7] All geometry structures were optimized with the 6-31G(d,p) basis set. On the basis of optimized geometries, the best binding structures of bilirubin with the repeating units of PAFs were searched with AutoDock Vina program,[8] in which all parameters are default. Following this, all binding structures were further optimized using B3LYP with 6-31G(d,p) basis set. To obtain more accurate energies, single-point calculations were performed based on these optimized geometries with the 6-311++G(2d,2p) basis set on all atoms. All binding energies (BE) were calculated to evaluate the adsorption strengths of bilirubin in the different PAFs. Basis set superposition error (BSSE) was considered in the calculations of binding energies.

Biocompatibility Experiments for iPAF-6

Cell viability and fluorescence images. The cells (L929 fibroblast cells or human umbilical vein endothelial cells (HUVECs)) were grown in Dulbecco's Modified

Eagle's medium (DMEM) supplemented with 10% fetal bovine serum (FBS) and 1% antibiotics (penicillin-streptomycin) at 37 °C in a humidified incubator containing 5% CO_2. A standard CCK-8 assay was used to assess the cytotoxicity of iPAF-6. Before the measurement, the iPAF-6 powders were sterilized with ethanol immersion and UV radiation. Cells at required density (1×10^5 cells $well^{-1}$) were seeded in 96 well plate and treated with iPAF-6 at varied concentrations (0.1, 0.3, 0.5, 1.0 and 2.0 mg mL^{-1}, respectively). Soon after treatment, plates were incubated for 24 h under above conditions. After washing with PBS thoroughly, 100 μL of CCK-8 was added into the culture medium and the resulted solution was incubated at 37 °C for 2 h. The negative control group was incubated with 200 μL culture medium DMEM instead of iPAF-6. The cell viability was determined by measuring the optical density at 450 nm on a microplate reader (Multiskan MK3) and calculated based on the following formula:

$$\text{Cell viability\%} = \frac{A_{\text{sample}}}{A_{\text{negative control}}} \times 100\%$$

where A_{sample} and $A_{negative\ control}$ are the absorptions at 450 nm for the experimental and negative control wells, respectively.

For fluorescence images, 5×10^4 cells $well^{-1}$ (L929 fibroblast or HUVEC cells) were incubated with 2.0 mg mL^{-1} of iPAF-6 powders for 3 days. After the incubation, cells were fixed using 4 % paraformaldehyde for 10 min and followed by the permeabilization in 0.1 % Triton X-100 for 5 min. After being washed with PBS three times, the cells were stained with TRITC Phalloidin (YESEN, China) and 4′,6-diamidino-2-phenylindole (DAPI, Sigma-aldrich). Finally, cells were washed three times with PBS and observed directly under Fluorescent Inverted microscope (FIM; iX53, OLYMPUS, Japen).

Preparation of blood samples. Fresh blood, obtained from a healthy wister Rat (Changchun Yis Experimental Animal Technology Co., Ltd., ethical permission letter number: 20180094), was mixed with the anticoagulant (3.8% citrate, with a 1:9 ratio of anticoagulant to blood) to get the fresh whole anticoagulant blood. All the animal experiments were conducted in compliance with the guidelines for the care and use of

laboratory animals from the National Institutes of Health, and authorize by the Ethics Committee of the Changchun University of Chinese Medicine. Platelet-poor plasma (PPP) was obtained by centrifuging the whole anticoagulant blood at 4000 rpm for 15 min.

Hemolysis Assay. Hemolytic ability was determined by incubating the iPAF-6 powders with red blood cells at 37 °C for 1 h. 1 mL rat blood was placed in the centrifuge tube and centrifuged at 3000 rpm for 10 min. The serum (upper layer) was discarded and the blood cells (under layer) were washed three times with PBS solution, and then diluted with 10 mL. iPAF-6 materials were washed with PBS and then iPAF-6 with different amounts (1.0, 3.0, 5.0, 10.0 and 20.0 mg, respectively) were added into the as-mentioned suspension. Deionized water and PBS were used as positive and negative control, respectively. After the incubation at 37 °C for 1 h, the mixture was centrifuged at 3000 rpm 10 min and the absorbance of the supernatant solution at 541 nm was recorded by UV-vis spectroscopy. The hemolysis rate was calculated using the following equation:

$$\text{Hemolysis rate (\%)} = \frac{\mathrm{OD_s} - \mathrm{OD_{nc}}}{\mathrm{OD_{pc}} - \mathrm{OD_{nc}}} \times 100\%$$

in which OD_s, OD_{pc} and OD_{nc} are the absorbances of the sample, positive control, and negative control, respectively.

Coagulation time tests. Activated partial time (APTT), thrombin time (TT) and prothrombin time (PT) were used to evaluate the anticoagulation property. Firstly, different amounts of iPAF-6 powders (1.0, 3.0, 5.0, 10.0 and 20.0 mg, respectively) were put into 24-well plate and the physiological saline were added into the plates for the immersion overnight, and then incubated at 37 °C for 1 h. After removing physiological saline, 100 μL fresh platelet-poor plasma (PPP) was introduced and incubated with the powders at 37 °C for 20 min. Platelet-poor plasma (PPP) could be obtained by centrifuging the whole anticoagulant blood at 4000 rpm for 15 min. For APTT measurement, 100 μL of APTT agent was added to the wells at 37 °C and

incubated at 37 °C for another 5 min. Then, 100 μL of 0.025 M $CaCl_2$ was added. Then, the coagulation time was measured as APTT value. For TT test, 100 μL of thrombin agent (incubated 10 min at 37 °C before use) was added to the wells, and then TT values were measured. To test PT, 100 μL Thromborel S (incubated 10 min before use) was added to the wells at 37 °C and further incubated at 37 °C for 2 min, and then the PT values were measured. Pure PPP without samples was the control group. All measurements were carried out six times.

Whole blood clotting test. The whole blood clotting test was performed according to the previous report.[9] Different amounts of iPAF-6 powders (0.5, 1.5, 2.5, 5.0 and 10.0 mg, respectively) were placed into 10 mL centrifuge tube, respectively. Then, 5.0 mL fresh whole anticoagulant blood was added to each sample and 0.5 mL of 0.2 M $CaCl_2$ solution was added instantly to initiate blood clotting response. The blood clotting time was observed and recorded at 37 °C.

Platelet activation test. The platelet activation was tested using an enzyme-linked immune sorbent assay with a Human Platelet Factor4 (PF4) kit. Different amounts of iPAF-6 (0.5, 1.5, 2.5, 5.0 and 10.0 mg, respectively) were immersed in PBS overnight. After removing the PBS, 5 mL of fresh whole blood was added. After being incubated at 37 °C for 1.5 hour, the supernatant whole blood was centrifuged for 10 minutes at 2500 rpm to obtain plasma. 40 μL of the obtained plasma was diluted for 10 times with PF4-Sample Diluent and then 200 μL of the diluted plasma was added into another Antibody Coated Well (provided by the PF4 kit). Finally, the detections were carried out according to the instruction manuals. Whole blood was used as control sample.

Complement activation test. Complement activation (C3a and C5a) was measured using enzymelinked immunosorbent assays. Fresh whole anticoagulant blood was centrifuged for 15min at 1500 rpm to obtain plasma. Then the plasma was incubated

with different concentrations of iPAF-6 (0.1, 0.3, 0.5, 1.0 and 2.0 mg mL^{-1}, respectively) for 2 h at 37 °C. A control experiment was conducted simultaneously using the same method without the addition of test samples. For the C3a test, 5 μL of the obtained plasma was diluted 500 times with C3a-sample diluent and 100 μL of the diluted plasma was then added to an antibody-coated well (provided by the C3a kit). For the C5a test, 10 μL of the obtained plasma was diluted 10 times with C5a-sample diluent and the diluted plasma was then added into another antibody-coated well (provided by the C5a kit). All the mentioned measurements were performed according to the instruction manuals.

Characterization

The morphology of obtained samples was observed with field emission scanning electron microscopy (SEM, Hitachi SU8010). Energy-dispersive X-ray spectroscopy (EDS) images of relevant samples were obtained from scanning electron microscopy. X-ray diffraction (XRD) measurements were carried out on a Rigaku SmartLab X-ray diffractometer with Cu-Kα radiation of λ=1.5418 Å (40 kV, 30 mA). 1H NMR spectra were recorded on Varian Unity Inova 600 MHz NMR spectrometer. Fourier-Transform infrared (FT-IR) spectra were acquired using a Nicolet iS50 Fourier transform infrared spectrometer. Thermogravimetric analysis (TGA) data were obtained on a Mettler toledo thermal analyzer at a heating rate of 10 °C min^{-1} under air atmosphere. Solid-state ^{13}C cross-polarization magic angle spinning nuclear magnetic resonance (CP/MAS NMR) measurement was performed on a Bruker Avance III model 400 MHz NMR spectrometer at a MAS rate of 5 kHz. The Elemental analysis (for C, H, and N) was measured using a Perkin Elmer 2400 Series II CHNS/O Analyzer. Analysis of the X-ray photo-electron spectra (XPS) was performed on Thermo ESCALAB 250 spectrometer with a Mg-K (1253.6 eV) achromatic X-ray source. The N_2 adsorption-desorption isotherms were measured on the Micromeritics ASAP 2010M analyzer. The concentrations of bilirubin in the solution with and without BSA were determined by an UV−vis spectrometer at the

wavelength of 460 nm and 438 nm, respectively.

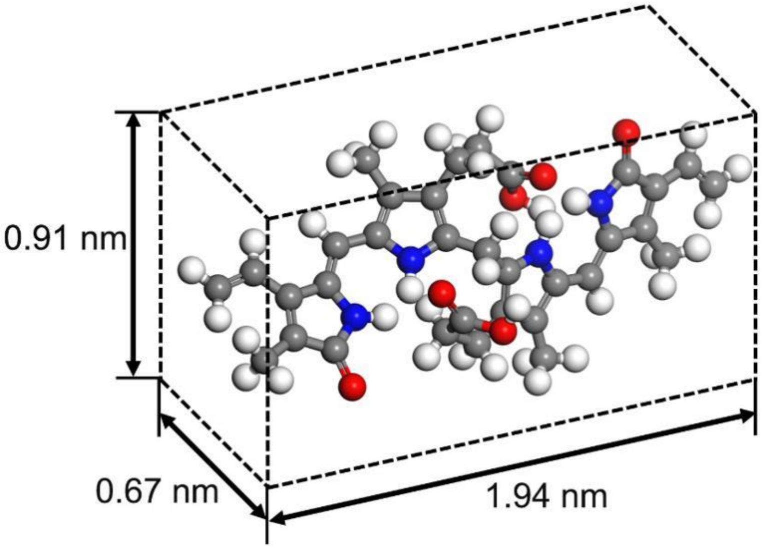

Figure S3. Dimensions of bilirubin from Material Studio (Mw = 584.67 g mol^{-1}).

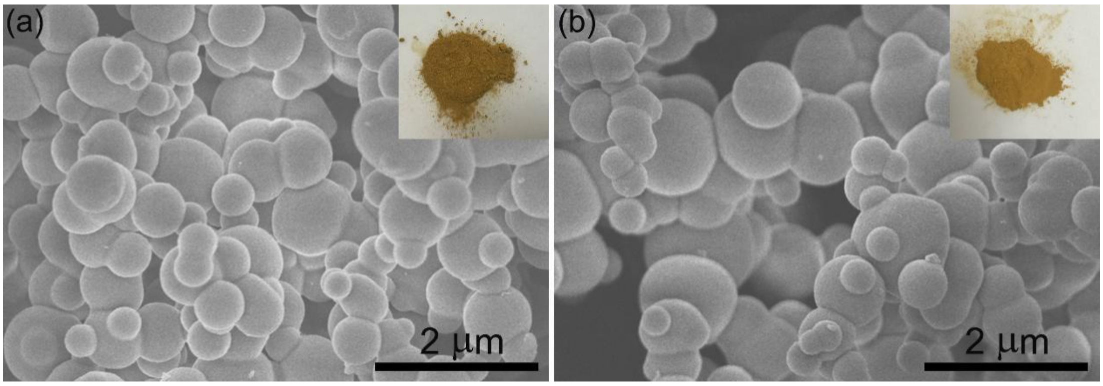

Figure S4. SEM images of (a) iPAF-5 and (b) iPAF-6 (insets are their digital photographs).

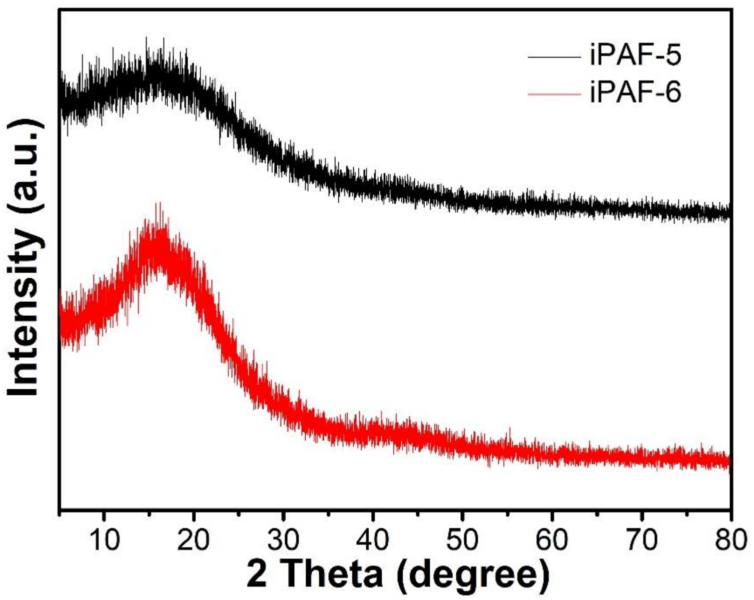

Figure S5. Powder X-ray diffraction (PXRD) patterns of iPAF-5 and iPAF-6.

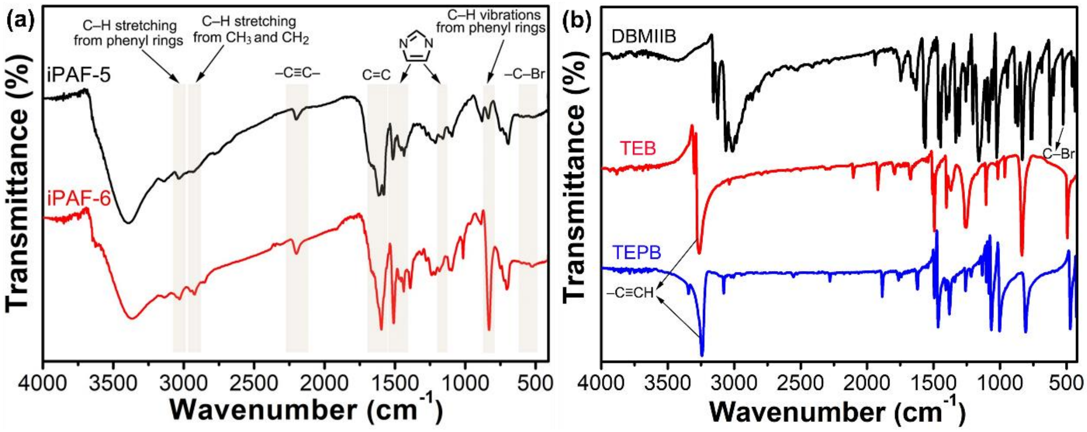

Figure S6. FTIR spectra of iPAF-5 and iPAF-6 (a), and the monomers for iPAF-5 and iPAF-6 (b).

As shown in Figure S6, the characteristic peaks of C−Br band from bromophenyl in DBMIIB at 500~600 cm^{-1} and–C≡CH from TEB (or TEPB) at 3270 cm^{-1} almost disappear in the FTIR spectra of iPAF-5 and iPAF-6.[10] Moreover, both spectra show the peaks of –C≡C– at 2200 cm^{-1},[11] indicating the cross-coupling reactions between DBMIIB and TEB (or TEPB). In addition, the absorption peaks of $–CH_2–$, $–CH_3$, phenyl and imidazolium rings also exist in the spectra.[12]

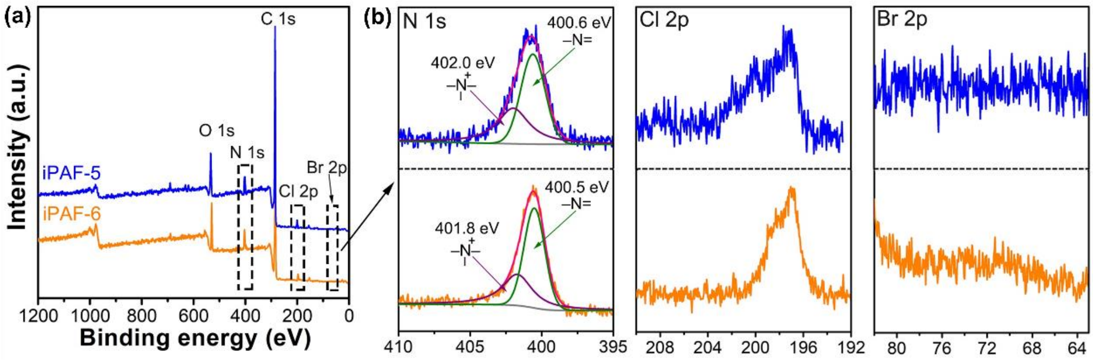

Figure S7. (a) XPS survey spectra of iPAF-5 and iPAF-6 and (b) their high-resolution XPS spectra of N 1s, Cl 2p and Br 2p.

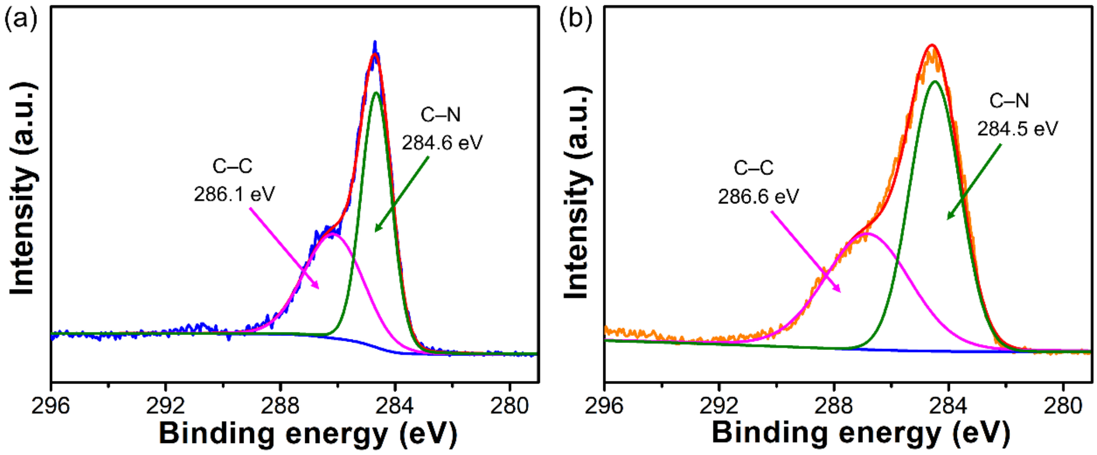

Figure S8. High-resolution XPS spectra of C 1s for (a) iPAF-5 and (b) iPAF-6.

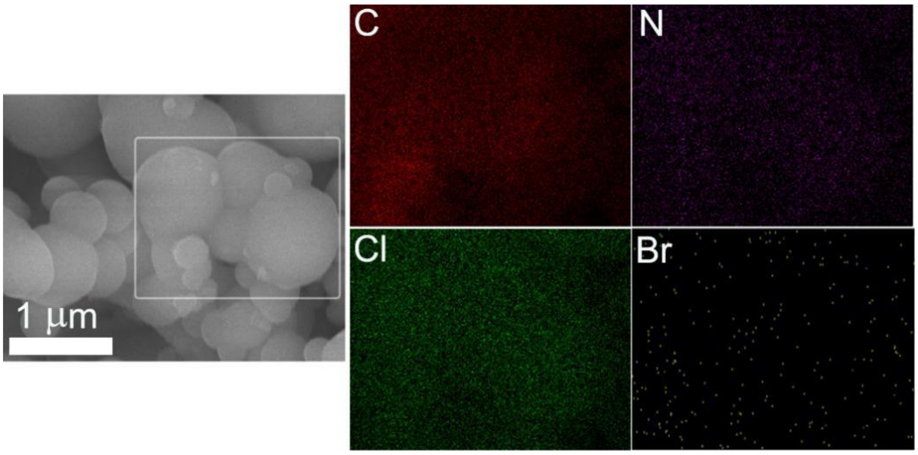

Figure S9. EDS mapping of iPAF-6 from the SEM image.

Table S1 Elemental analysis results (C, H and N) for iPAF-5 and iPAF-6.

Sample		C wt%	H wt%	N wt%
iPAF-5	Calcd for $C_{57}H_{39}N_6Cl_3$	74.88	4.27	9.20
	Found	68.70	4.75	8.75
iPAF-6	Calcd for $C_{93}H_{63}N_6Cl_3$	81.50	4.60	6.13
	Found	76.61	4.94	5.97

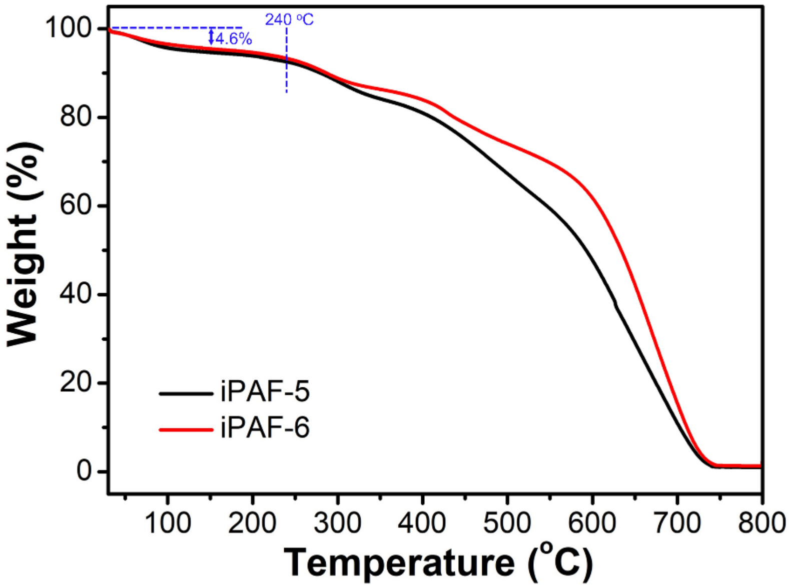

Figure S10. TGA curves of iPAF-5 and iPAF-6 under air.

The initial weight losses before 150 ^{o}C for both samples are attributed to the water evaporation and both iPAF-5 and iPAF-6 are thermally stable up to 240 ^{o}C in air atmosphere.

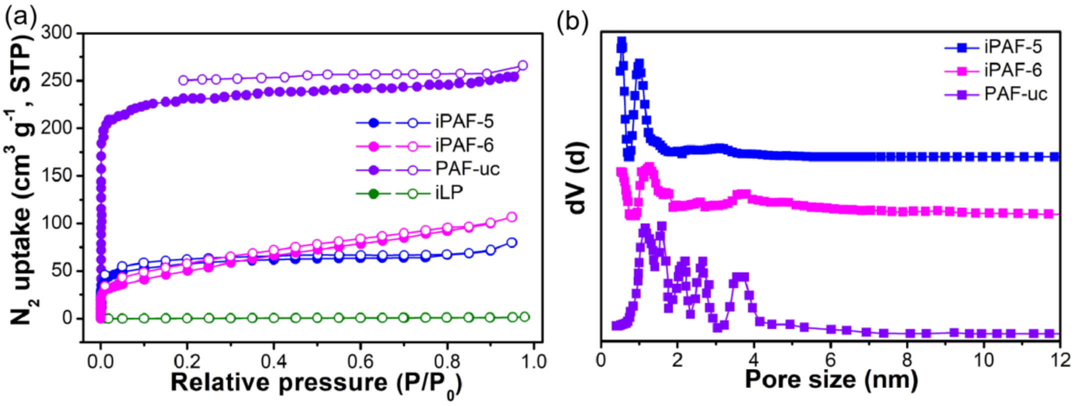

Figure S11. (a) Nitrogen adsorption isotherms and (b) pore size distributions based on NLDFT of the prepared samples.

Table S2 Textural data of all related materials from nitrogen adsorption.

Sample	S_{BET} ($m^2\ g^{-1}$)	V_{total} ($cm^3\ g^{-1}$)[a]	V_{micro} ($cm^3\ g^{-1}$)[b]	V_{meso} ($cm^3\ g^{-1}$)[c]
iPAF-5	208	0.12	0.09	0.03
iPAF-6	148	0.16	0.05	0.11
PAF-uc	561	0.29	0.13	0.16
iLP	1.8	--	--	--

[a]Total pore volumes were calculated at P/P_0 = 0.98. [b]Cumulative micropore volumes were calculated at pore width r ≤ 2.0 nm. [c]Cumulative mesopore volumes were equal to V_{total} - V_{micro}.

Table S3 Elemental analysis results (C, H and N) for PAF-uc and iLP.

Sample		C wt%	H wt%	N wt%
PAF-uc	Calcd for $C_{39}H_{21}$	95.71	4.29	--
	Found	88.64	5.10	--
iLP	Calcd for $C_{21}H_{15}N_2Cl$	76.25	4.54	8.47
	Found	73.53	5.32	8.17

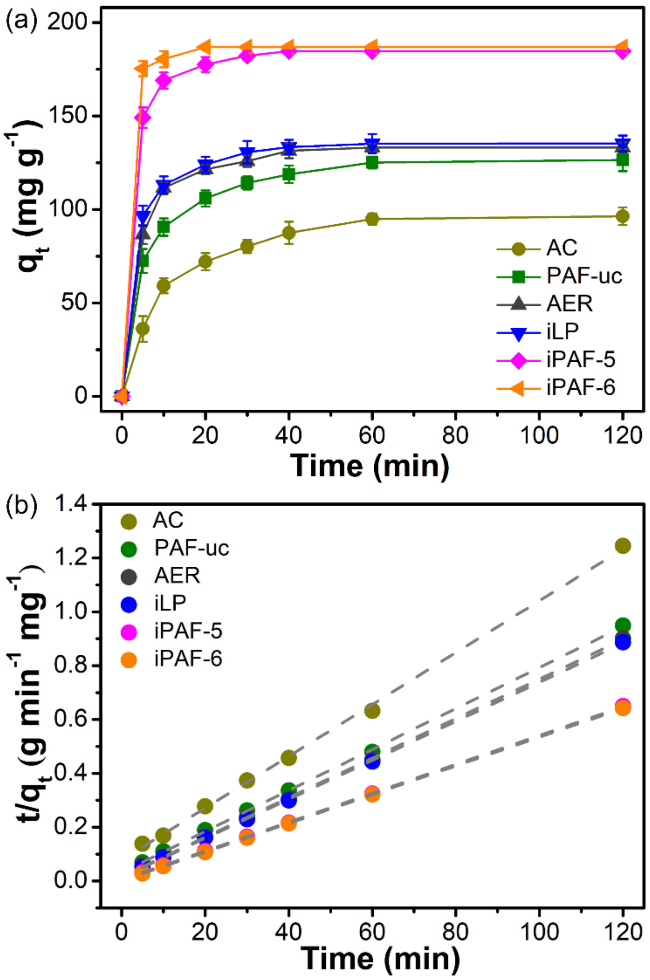

Figure S12. (a) Adsorption kinetics of different tested adsorbents toward bilirubin and (b) their pseudo-second-order linear fitting plots.

Table S4 Kinetics parameters from the pseudo-second-order model.

Adsorbent	Pseudo-second-order model		
	q_e (mg g^{-1})	k_2 (g mg^{-1} min^{-1})	R^2
AC	103.84	1.18×10^{-3}	0.9986
PAF-uc	131.41	1.77×10^{-3}	0.9955
AER	136.24	3.28×10^{-3}	0.9997
iLP	137.93	3.84×10^{-3}	0.9998
iPAF-5	186.56	6.09×10^{-3}	0.9999
iPAF-6	187.27	2.72×10^{-2}	0.9999

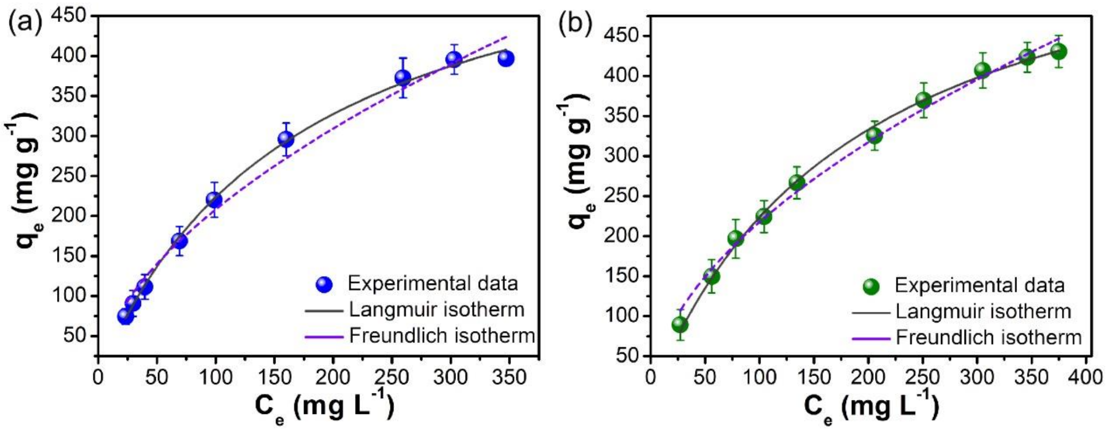

Figure S13. Bilirubin adsorption isotherms by (a) iLP and (b) PAF-uc analyzed through Langmuir model and Freundlich model.

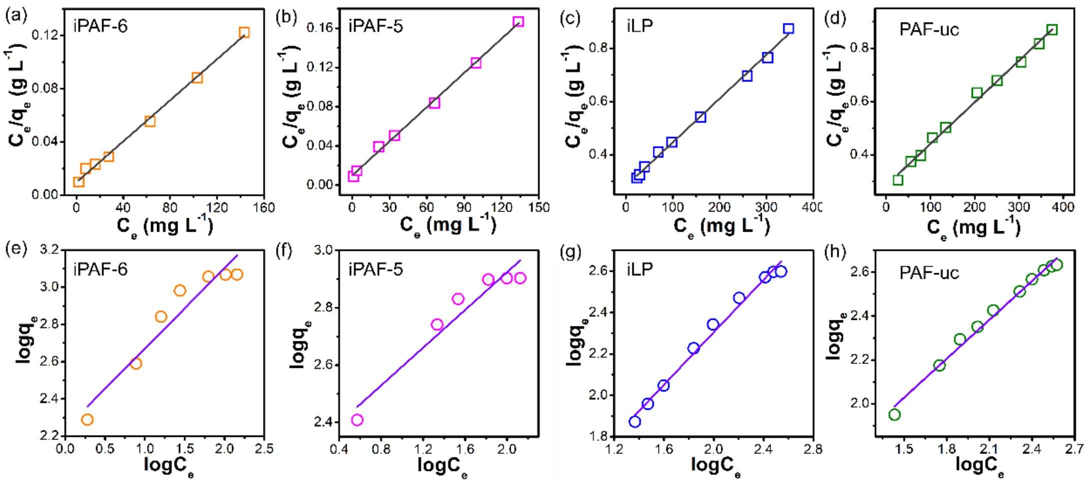

Figure S14. Langmuir linear plots (a~d) and Freundlich linear plots (e~h) for iPAF-6, iPAF-5, iLP and PAF-uc

Table S5 Langmuir and Freundlich parameters for bilirubin adsorption by different adsorbents.

Adsorbent	Langmuir isotherm			Freundlich isotherm		
	q_m (mg g^{-1})	b (L mg^{-1})	R^2	K_F	n	R^2
iPAF-6	1296.34	0.0799	0.9953	172.98	2.31	0.9086
iPAF-5	862.07	0.118	0.9927	184.50	3.04	0.9168
iLP	556.04	0.00585	0.9952	10.91	1.58	0.9869
PAF-uc	641.03	0.00548	0.9940	13.80	1.69	0.9883

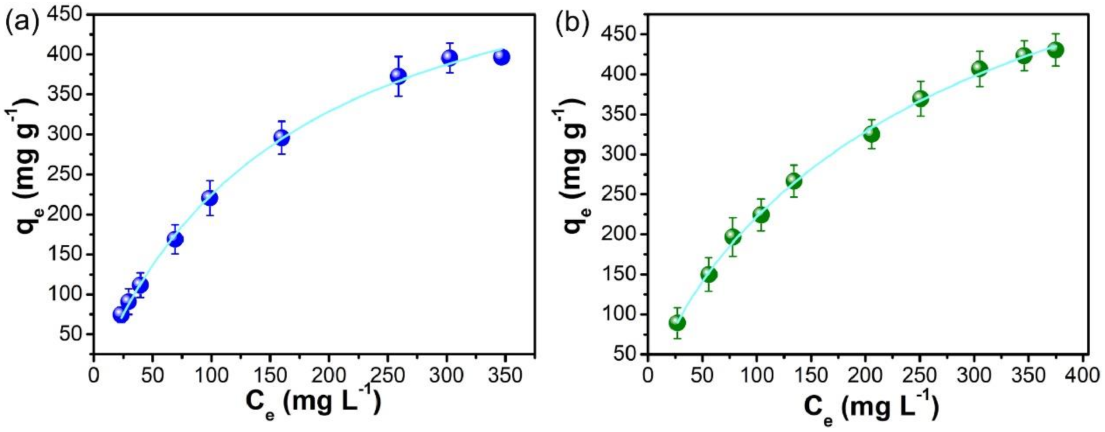

Figure S15. Bilirubin adsorption isotherms by (a) iLP and (b) PAF-uc analyzed through Zhu and Gu isotherm model.

Table S6 Zhu and Gu isotherm parameters for bilirubin adsorption by different adsorbents.

	Zhu and Gu isotherm model				
Adsorbent	q_m (mg g^{-1})	K_1	K_2	m	R^2
iPAF-6	1248.68	0.626	0.00122	3.4	0.9999
iPAF-5	832.54	0.796	0.00429	2.8	0.9969
iLP	561.38	0.0130	0.00601	2.0	0.9972
PAF-uc	644.82	0.0157	0.000493	2.3	0.9977

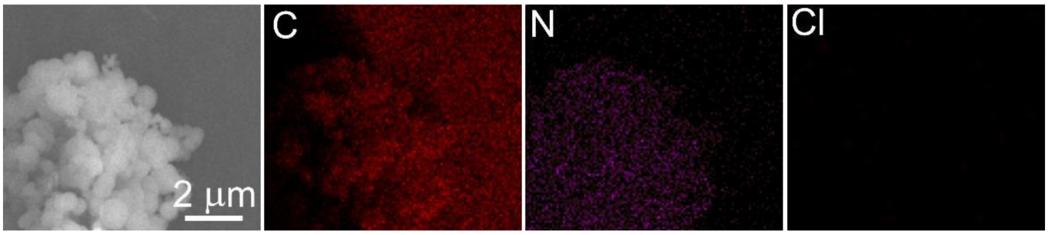

Figure S16. EDS mapping of iPAF-6 after bilirubin adsorption from the SEM image.

Table S7 Comparison of bilirubin adsorption results in the presence of interference factors for various adsorbents.

Materials	BLB conc. (mg L^{-1})[a]	Adsorbent dosage (mg mL^{-1})	Adsorption environment	T (°C)	Uptake (mg g^{-1})	Ref.
PDA-ordered mesoporous carbon	10~500	1.0	40 g L^{-1} BSA	–	122.7	[13]
Chitosan(CS)/SiO_2-graphene	200	2.3	50 g L^{-1} BSA	37	32.2	[14]
bPEI-PAN fibers	40~400	0.4~1.0	50 g L^{-1} BSA	37	112.9	[15]
3D nanofiber sponge	50~400	10.0	plasma	–	25.3	[16]
Clinical activated carbon	200	5.0	plasma	37	0.8	[17]
CS/amino-WCNT	150	–	15 g L^{-1} BSA	37	7.6	[18]
Lysine-chitin/CNT microspheres	200	1.3	10 g L^{-1} BSA	30	33.5	[19]
Heparin-modified CS/GO	306	6.3	serum	37	9.0	[20]
PCB-H103	150	3.0	serum	25	6.6	[21]
PCB-H103	150	3.0	40 g L^{-1} BSA	25	20.8	[21]
Porous graphene	20~100	0.1	40 g L^{-1} BSA	37	126.1	[22]
HAS-mPHEMA	206	1.0	plasma	25	64.7	[23]
p(GMA–MMA)-PEI	100	–	serum	25	23.6	[24]
N-doped porous carbon	50~300	0.3	40 g L^{-1} BSA	25	105.5	[25]
$CaCO_3$/polystyrene naocomposite	150	–	15 g L^{-1} BSA	37	25.0	[26]
Amine-PVA-co-PE membrane	200	–	50 g L^{-1} BSA	37	36.0	[27]
Imprinted poly(HEMA-MAT)	–	12.5	plasma	25	3.4	[28]
Powdered activated carbon	150	6.0	40 g L^{-1} BSA	25	8.0	[29]
rGO@HepMBm Nanocomposite	300	–	10 g L^{-1} BSA	37	166.3	[30]
iPAF-6	200	1.0	50 g L^{-1} BSA	37	192.3	This work

[a]Single concentration values denote experiments which were performed at a single bilirubin concentration. Concentration range values denote the bilirubin concentration range of the solution in contact with the adsorbent at equilibrium.

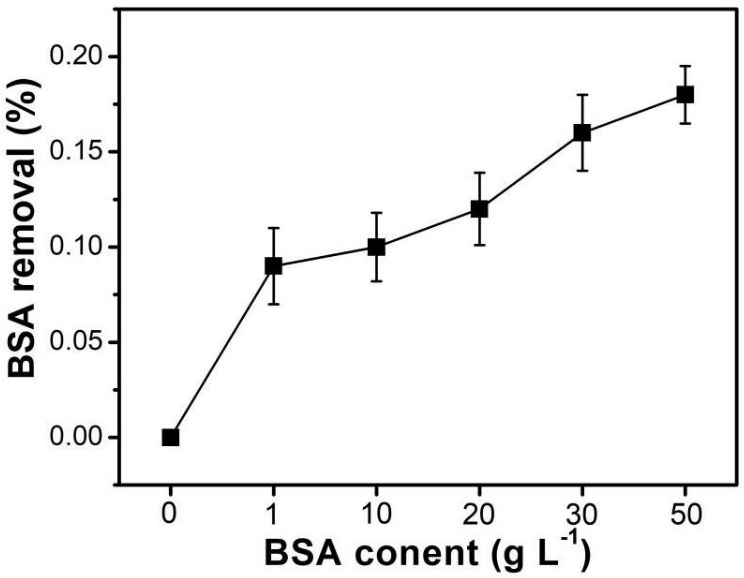

Figure S17. BSA removal during the influence of albumin on bilirubin adsorption processes.

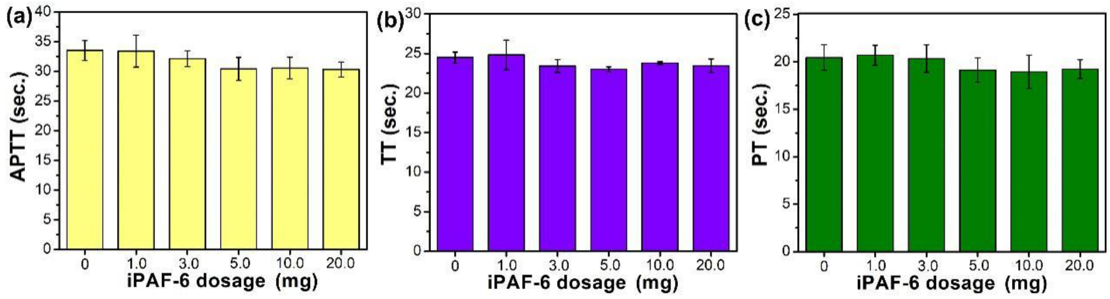

Figure S18. (a) APTT, (b) TT and (c) PT values for different amounts of iPAF-6.

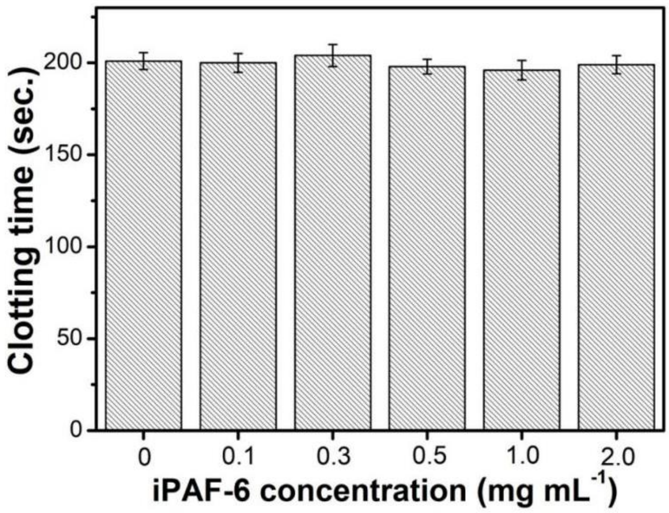

Figure S19. Whole blood clotting time of iPAF-6 with different concentrations.

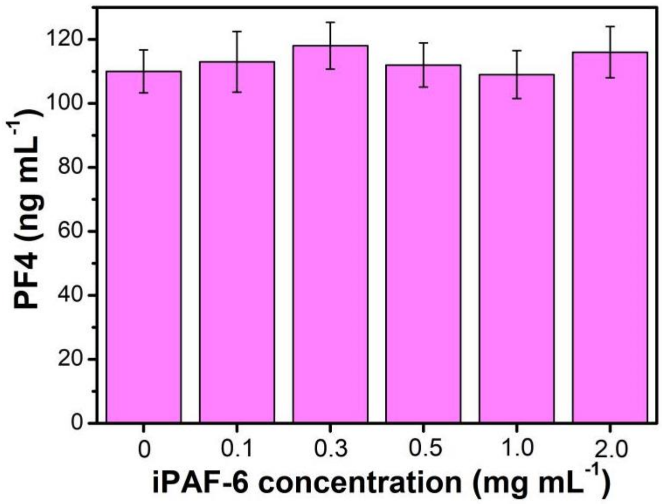

Figure S20. Concentrations of PF4 in whole blood after incubation with different amounts of iPAF-6.

Previous study showed that foreign materials could induce the activation of platelets when contacted with blood, which would lead to the release of PF4 and initiate some other coagulation factors, and accelerate the formation of coagulations and thrombin.[31,32] Thus, we used PF4 concentration level to evaluate the platelet activation for iPAF-6.

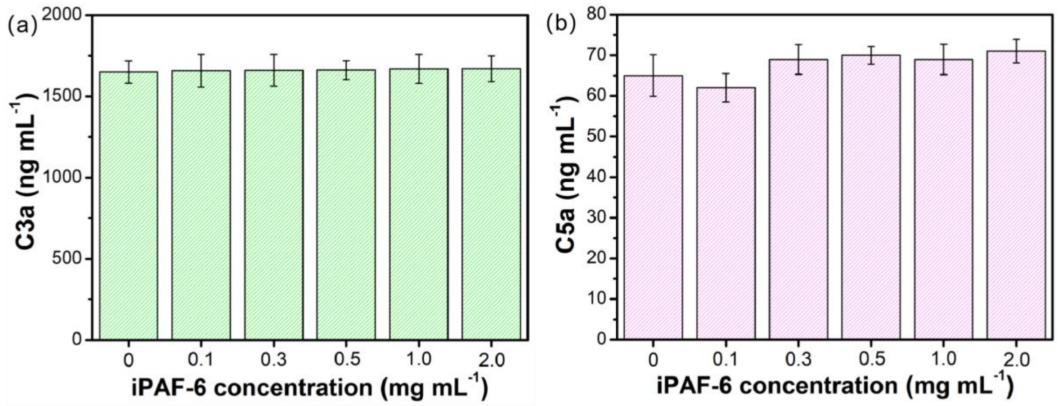

Figure S21. Concentrations of C3a (a) and C5a (b) in whole blood after incubation with different amounts of iPAF-6.

Complement activation is considered as a trigger of the host defense mechanism, which is generated by localized inflammatory mediator. Complement activation can also reflect the blood compatibility of the materials. C3a and C5a are the activation products of complement system, which are usually used to evaluate complement activation.[33,34]

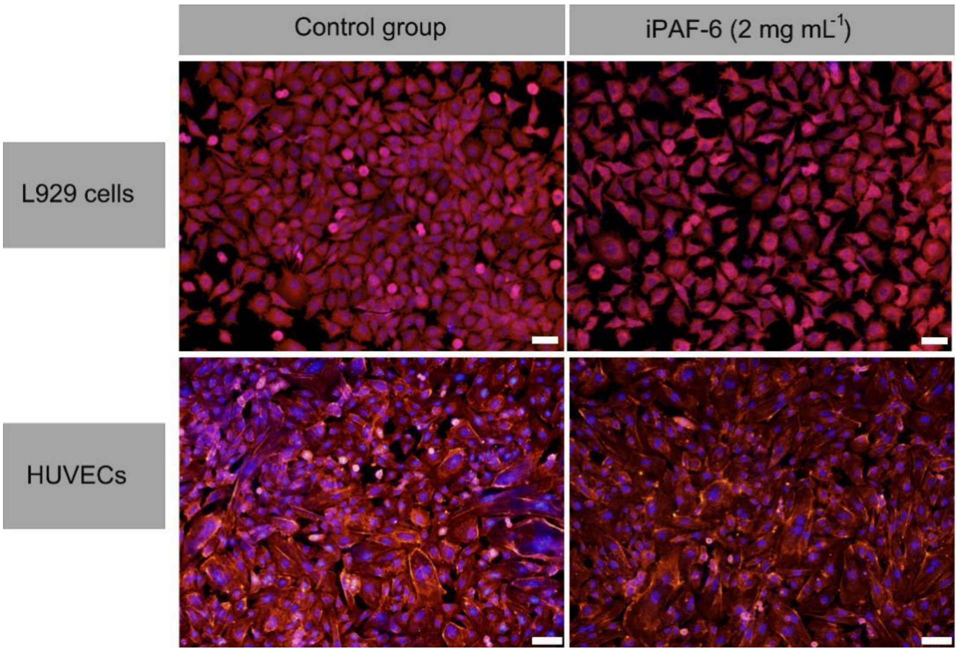

Figure S22. Fluorescence images of L929 fibroblast cells and HUVECs incubated with control group and 2 mg mL^{-1} iPAF-6 after 3 days (the inset bars represent 100 μm).

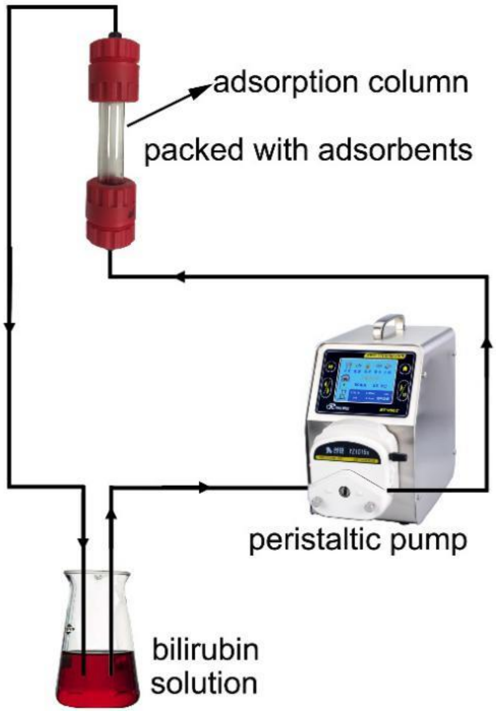

Figure S23. Schematic diagram for the typical laboratory-made hemoperfusion process.

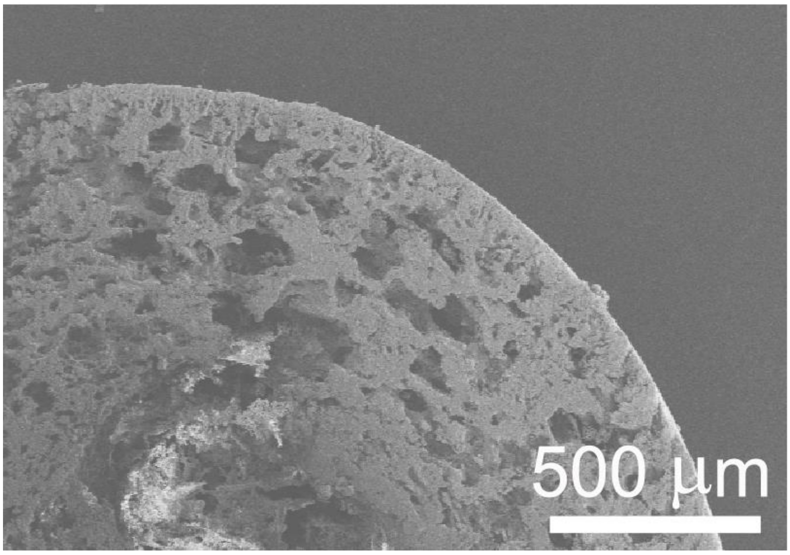

Figure S24. SEM image of iPAF-6/PES bead.

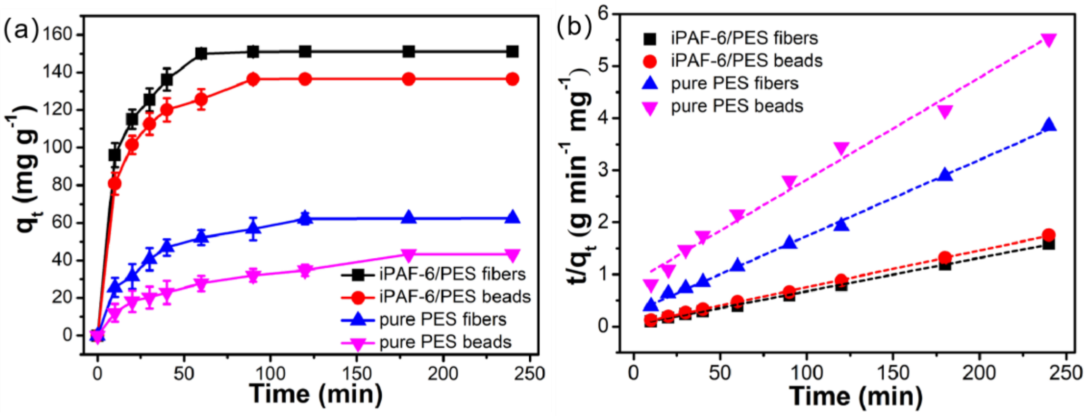

Figure S25. (a) Adsorption kinetics of bead or fiber adsorbents toward bilirubin and (b) their pseudo-second-order linear fitting plots.

Table S8 Kinetics parameters from the pseudo-second-order model of bilirubin adsorption by bead or fiber adsorbents.

Adsorbent	Pseudo-second-order model		
	q_e (mg g^{-1})	k_2 (g mg^{-1} min^{-1})	R^2
Pure PES fibers	68.25	7.91×10^{-4}	0.9978
Pure PES beads	50.99	4.47×10^{-4}	0.9851
iPAF-6/PES fibers	155.52	1.22×10^{-3}	0.9993
iPAF-6/PES beads	141.64	1.03×10^{-3}	0.9994

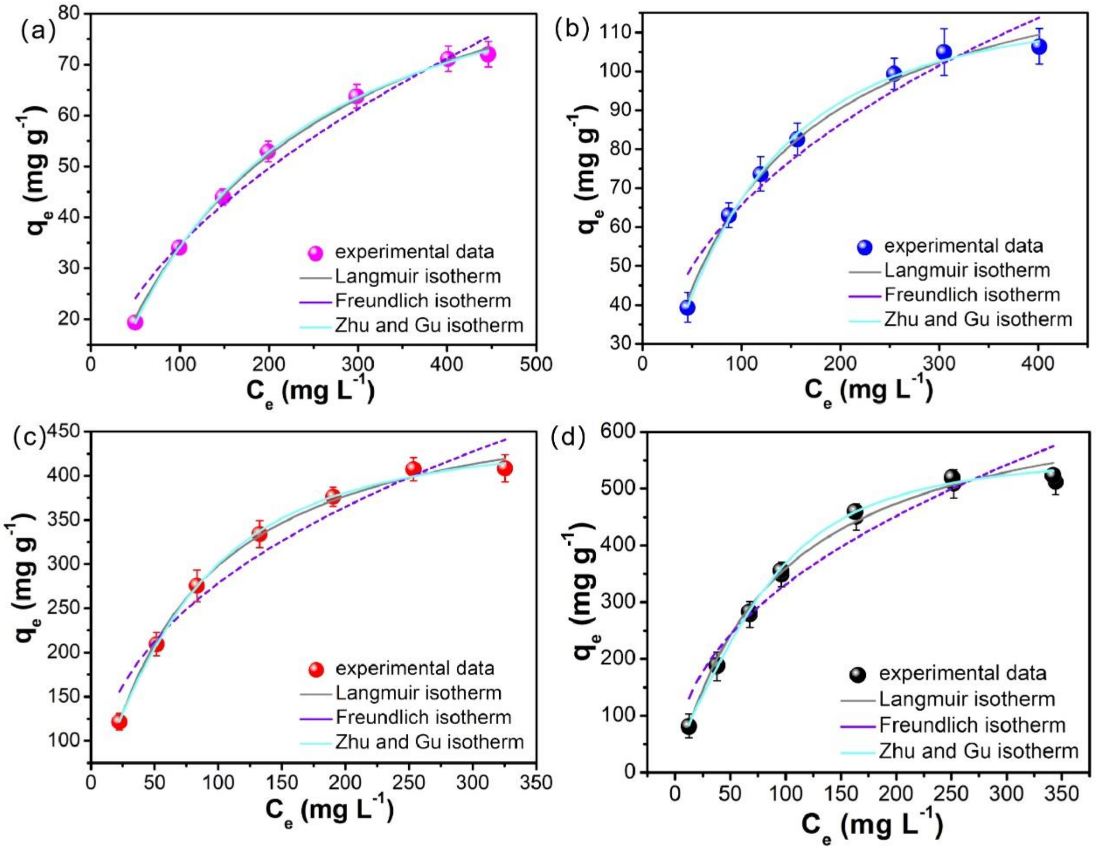

Figure S26. Bilirubin adsorption isotherms by (a) pure PES beads, (b) pure PES fibers, (c) iPAF-6/PES beads, and (d) iPAF-6/PES fibers analyzed through Langmuir model, Freundlich model, and Zhu and Gu isotherm model.

Table S9 Langmuir and Freundlich parameters for bilirubin adsorption by bead or fiber adsorbents

adsorbent	Langmuir isotherm			Freundlich isotherm		
	q_m (mg g^{-1})	b (L mg^{-1})	R^2	K_F	n	R^2
Pure PES fibers	138.53	0.00940	0.9926	10.58	2.52	0.9390
Pure PES beads	91.74	0.00462	0.9979	3.12	1.68	0.9718
iPAF-6/PES fibers	593.68	0.0107	0.9915	52.06	2.23	0.9350
iPAF-6/PES beads	482.20	0.0139	0.9960	46.30	2.56	0.9418

Table S10 Zhu and Gu isotherm parameters for bilirubin adsorption by bead or fiber adsorbents.

Adsorbent	Zhu and Gu isotherm model				
	q_m (mg g^{-1})	K_1	K_2	m	R^2
Pure PES fibers	118.65	0.0326	0.000778	2.5	0.9955
Pure PES beads	83.46	0.0132	0.000429	2.9	0.9987
iPAF-6/PES fibers	562.23	0.0519	0.000272	2.8	0.9975
iPAF-6/PES beads	448.76	0.0603	0.000875	2.6	0.9979

References

[1] Y. Tian, J. Song, Y. Zhu, H. Zhao, F. Muhammad, T. Ma, M. Chen, G. Zhu, *Chem. Sci*. **2019**, *10*, 606.

[2] B. Li, Y. Zhang, D. Ma, Z. Shi, S. Ma, *Nat. Commun*. **2014**, *5*, 5537.

[3] R. Zhao, Y. Tian, S. Li, T. Ma, H. Lei, G. Zhu, *J. Mater. Chem. A*, **2019**, *7*, 22559.

[4] a) R. M. Abdelhameed, H. Abdel-Gawad, C. M. Silva, J. Rocha, B. Hegazi1, A. M. S. Silva, *Int. J. Environ. Sci. Technol*. **2018**, *15*, 2283; b) P. Rekha, R. Muhammad, V. Sharma, M. Ramtekeb, P. Mohanty, *J. Mater. Chem. A* **2016**, *4*, 17866.

[5] A. D. Becke, *Phys. Rev. A* **1988**, *38*, 3098.

[6] C. Lee, W. Yang, R. G. Parr, *Phys. Rev. B* **1988**, *37*, 785.

[7] M. J. Frisch, G. W. Trucks, H. B. Schlegel, G. E. Scuseria, M. A. Robb, J. R. Cheeseman, G. Scalmani, V. Barone, B. Mennucci, G. A. Petersson, et al., Gaussian 09, Gaussian, Inc., Wallingford, CT, USA, **2009**.

[8] O. Trott, A. J. Olson, *J. Comput. Chem*. **2010**, *31*, 455.

[9] S. Hu, S. C. Bi, D. Yan, Z. Z. Zhou, G. H. Sun, X. J. Cheng, X. G. Chen, *Carbohydr. Polym*. **2018**, *184*, 154.

[10] a) B. Liang, H. Wang, X. Shi, B. Shen, X. He, Z. A. Ghazi, N. A. Khan, H. Sin, A. M. Khattak, L. Li, Z. Tang, *Nature Chem*. **2018**, *10*, 961; b) J. Chen, W. Yan, E. J.

Townsend, J. Feng, L. Pan, V. D. A. Hernandez, C. F. J. Faul, *Angew. Chem. Int. Ed.* **2019**, *58*, 1.

[11] Z. Yan, Y. Yuan, Y. Tian, D. Zhang, G. Zhu, *Angew. Chem. Int. Ed*. **2015**, *54*, 12733.

[12] a) Y. Yuan, Y. Yang, M. Faheem, X. Zou, X. Ma, Z. Wang, Q. Meng, L. Wang, S. Zhao, G. Zhu, *Adv. Mater*. **2018**, *30*, 1800069; b) Y. Zhang, G. Chen, L. Wu, K. Liu, H. Zhong, Z. Long, M. Tong, Z. Yang, S. Dai, *Chem. Commun*. **2020**, *56*, 3309.

[13] S. Huang, J. Zheng, Y. Zhang, J. Zheng, Z. Zhuang, Q. Yang, F. Wang, G. Chen, S. Huang, G. Ouyang, *J. Mater. Chem. B,* **2020**, *8*, 290.

[14] J. Chen, Y. Ma, L. Wang, W. Han, Y. Chai, T. Wang, J. Li, L. Ou, *Carbon* **2019**, *143*, 352.

[15] R. Zhao, Y. Li, X. Li, Y. Li, B. Sun, S. Chao, C. Wang, *J. Colloid Interface Sci*. **2018**, *514*, 675.

[16] Z. Yuan, Y. Lia, D. Zhao, K. Zhang, F. Wang, C. Wang, Y. Wen, *Colloids and surfaces. B. Biointerfaces* **2018**, *172,* 161.

[17] G. Tao, L. Zhang, Z. Hua, Y. Chen, L. Guo, J. Zhang, Z. Shu, J. Gao, H. Chen, W. Wu, Z. Liu, J. Shi, *Carbon* **2014**, *66*, 547.

[18] W. Zong, J. Chen, W. Han, J. Chen, Y. Wang, W. Wang, G. Cheng, L. Ou, Y. Yu, *J. Biomed. Mater. Res. B* **2018**, *106*, 96.

[19] S.Wu, B. Duan, X. Zeng, A. Lu, X. Xu, Y. Wang, Q. Ye, L. Zhang, *J. Mater. Chem. B* **2017**, *5*, 2952.

[20] H. Wei, L. Han, Y. Tang, J. Ren, Z. Zhao, L. Jia, *J. Mater. Chem. B* **2015**, *3*, 1646.

[21] Q. Li, J.Yang, N. Cai, J. Zhang, T. Xu, W. Zhao, H. Guo, Y. Zhu, L. Zhang, *J. Colloid Interface Sci.* **2019**, *555,* 145.

[22] C. F. Ma, Q. Gao, K. S. Xia, Z. Y. Huang, B. Han, C. G. Zhou, *Colloids surfaces. B. Biointerfaces* **2017**, *149,* 146.

[23] A. Y. Rad, H. Yavuz, M. Kocakulak, A. Denizli, *Macromol. Biosci*. **2003**, *3*, 471.

[24] M. Y. Arca, E. Yaln, B. Gülay, *Polym. Int*. **2005**, *54*, 153.

[25] C. F. Ma, Q. Gao, J. Zhou, Q. X. Chen, B. Han, K. S. Xia, C. G. Zhou, *RSC Adv.* **2017**, *7,* 2081.

[26] J. Chen, G. Cheng, Y. Chai, W. Han, W. Zong, J. Chen, C. Li, W. Wang, L. Ou, Y, Yu, *Colloids Surfaces. B. Biointerfaces* **2018**, *161*, 480.

[27] W. Wang, H. Zhang, Z. Zhang, M. Luo, Y. Wang, Q. Liu, Y. Chen, M. Li, D. Wang, *Colloids Surfaces. B. Biointerfaces* **2017**, *150*, 271.

[28] G. Baydemir, M. Andac, N. Bereli, R. Say, A. Denizli, *Ind. Eng. Chem. Res*. **2007**, *46*, 2843.

[29] N. Cai, Qi. Li, J. Zhang, T. Xu, W. Zhao, J. Yang, L. Zhang, *J. Colloid Interface Sci.* **2017**, *503*, 168.

[30] X. Song, T. Xu, L. Yang, Y. Li, Y. Yang, L. Jin, J. Zhang, R. Zhong, S. Sun, W. Zhao, C. Zhao, *Biomacromolecules* **2020**, *21*, 1762.

[31] Y. Li, M. Han, Y. Wang, Q. Liu, W. Zhao, B. Su, C. Zhao, *Carbohyd. Polym.* **2018**, *202*, 116.

[32] M. B. Gorbet, M. V. Sefton, *Biomaterials* **2004**, *25*, 5681.

[33] D. Ricklin, E. S. Reis, J. D. Lambris, *Nat. Rev. Nephrol.* **2016**, *12*, 383.

[34] X. Huang, R. Wang, T. Lu, D. Zhou, W. Zhao, S. Sun, C. Zhao, *Biomacromolecules* **2016**, *17*, 4011.
